# CK1ε and p120‐catenin control Ror2 function in noncanonical Wnt signaling

**DOI:** 10.1002/1878-0261.12184

**Published:** 2018-03-14

**Authors:** Josué Curto, Beatriz Del Valle‐Pérez, Aida Villarroel, Guillem Fuertes, Meritxell Vinyoles, Raúl Peña, Antonio García de Herreros, Mireia Duñach

**Affiliations:** ^1^ Departament de Bioquímica i Biologia Molecular CEB Facultat de Medicina Universitat Autònoma de Barcelona Bellaterra Spain; ^2^ Programa de Recerca en Càncer Institut Hospital del Mar d'Investigacions Mèdiques (IMIM) Barcelona Spain; ^3^ Departament de Ciències Experimentals i de la Salut Universitat Pompeu Fabra Barcelona Spain

**Keywords:** CK1ε, noncanonical Wnt, p120‐catenin, Ror2

## Abstract

Canonical and noncanonical Wnt pathways share some common elements but differ in the responses they evoke. Similar to Wnt ligands acting through the canonical pathway, Wnts that activate the noncanonical signaling, such as Wnt5a, promote Disheveled (Dvl) phosphorylation and its binding to the Frizzled (Fz) Wnt receptor complex. The protein kinase CK1ε is required for Dvl/Fz association in both canonical and noncanonical signaling. Here we show that differently to its binding to canonical Wnt receptor complex, CK1ε does not require p120‐catenin for the association with the Wnt5a co‐receptor Ror2. Wnt5a promotes the formation of the Ror2–Fz complex and enables the activation of Ror2‐bound CK1ε by Fz‐associated protein phosphatase 2A. Moreover, CK1ε also regulates Ror2 protein levels; CK1ε association stabilizes Ror2, which undergoes lysosomal‐dependent degradation in the absence of this kinase. Although p120‐catenin is not required for CK1ε association with Ror2, it also participates in this signaling pathway as p120‐catenin binds and maintains Ror2 at the plasma membrane; in p120‐depleted cells, Ror2 is rapidly internalized through a clathrin‐dependent mechanism. Accordingly, downregulation of p120‐catenin or CK1ε affects late responses to Wnt5a that are also sensitive to Ror2, such as *SIAH2* transcription, cell invasion, or cortical actin polarization. Our results explain how CK1ε is activated by noncanonical Wnt and identify p120‐catenin and CK1ε as two critical factors controlling Ror2 function.

**Abbreviations**
Dapi: 4',6‐diamidino‐2‐phenylindole
DMEM: Dulbecco's modified Eagle's medium
Dvl: Disheveled
Fz: Frizzled
GST: glutathione S‐transferase
Hb: herbimycin
MDC: monodansylcadaverine
MSCs: mesenchymal stem cells
PEI: polyethylenimine
PP2A: protein phosphatase 2A
RT‐PCR: reverse transcription polymerase chain reaction
WB: Western blot

## 1 Introduction

Wnts are secreted proteins that induce the activation of several signaling pathways essential in different physiological processes. According to their effect on β‐catenin, they have been classified as canonical and noncanonical Wnts. Canonical Wnts, such as Wnt3a, enhance β‐catenin stability, translocate this protein to the nucleus, and increase β‐catenin transcriptional activity inducing the expression of different target genes (MacDonald and He, 2013). In contrast, the noncanonical Wnts, such as Wnt5a, activate β‐catenin‐independent pathways involving the small GTPases Rho and Rac1, Vangl2 phosphoprotein, and the protein kinases JNK2, ERK2, and Ca^2+^/calmodulin‐dependent kinase (Gao *et al*., 2011; Kikuchi *et al*., 2012). This β‐catenin‐independent, noncanonical Wnt pathway is associated with the control of cytoskeleton, cell migration, and polarity (Kikuchi *et al*., 2012).

Activation of the noncanonical pathway by Wnt5a has been shown to be involved in tumor growth and invasion (Ford *et al*., 2013). Intriguingly, the effects observed by different authors appear to be totally contrary to each other: while some conclude that Wnt5a and its co‐receptor Ror2 are involved in epithelial‐to‐mesenchymal transition, invasion, and metastasis (Gujral *et al*., 2014; Ren *et al*., 2011; Shojima *et al*., 2015), others conclude that Wnt5a inhibits metastasis (Jiang *et al*., 2013; Säfholm *et al*., 2008) and block expansion of tumor‐initiating cells (Borcherding *et al*., 2015). To better understand the reasons for these apparent discrepancies, we have analyzed the early molecular events triggered by Wnt5a and compared these events with those induced by canonical Wnt.

The canonical Wnt pathway has been extensively studied. The cellular receptor complex for these Wnt ligands is composed by the Wnt high‐affinity transmembrane protein Frizzled (Fz) and the co‐receptors LRP5 or LRP6, which are two highly homologous proteins (referred to herein as LRP5/6) (MacDonald and He, 2013). Binding of Wnt ligands promotes the association of Fz with LRP5/6, and the rapid activation of CK1ε (Casagolda *et al*., 2010; Swiatek *et al*., 2004). We have previously described that LRP5/6 is constitutively bound to CK1ε through their mutual interactions with N‐ or E‐cadherin and p120‐catenin, respectively (Casagolda *et al*., 2010; Hay *et al*., 2009). Assembly of the Fz–LRP5/6 receptor complex facilitates dephosphorylation of the LRP5/6 and p120‐catenin‐bound CK1ε as well as its activation by the Fz‐associated protein phosphatase 2A (PP2A) (Vinyoles *et al*., 2017). PP2A binding requires the interaction with Fz of the PP2A‐regulatory subunit PR61ε (also known as B56 or B′), the product of *PPP2R5E* gene (Vinyoles *et al*., 2017). Highlighting the relevance of PR61ε in this pathway, deletion of this gene product interferes with Wnt signaling in *Xenopus* early embryogenesis, upstream of Dvl2 (Yang *et al*., 2003).

Once activated, CK1ε enhances the association of Dvl2 with Fz by phosphorylating either Dvl2, Fz, or both (Duñach *et al*., 2017; MacDonald and He, 2013). The interaction with Dvl2 enables further reactions, such as recruitment of CK1γ, the LRP5/6 phosphorylation by CK1γ on Thr1479 and other residues (MacDonald and He, 2013), and the binding to the complex of Axin and the associated protein kinases CK1α and GSK3α and β (Bilic *et al*., 2007; Del Valle‐Pérez *et al*., 2011). Association of Axin correlates with the inhibition of GSK3 activity on β‐catenin Ser37, therefore preventing polyubiquitination by β‐TRCP1 ubiquitin ligase and β‐catenin proteasomal degradation, and facilitating β‐catenin/Tcf4 transcriptional activity (Duñach *et al*., 2017; MacDonald and He, 2013). Moreover, activation of other downstream elements, such as JNK2, facilitates the traffic of stabilized β‐catenin to the nucleus (Wu *et al*., 2008).

Wnts that activate the noncanonical pathway, such as Wnt5a, also use Fz proteins as cellular receptors; however, they do not bind to LRP5/6 (Kikuchi *et al*., 2012). Instead, they interact with Ror2, a transmembrane tyrosine kinase that also contains an extracellular cysteine‐rich domain, the binding site for Wnt proteins (Green *et al*., 2014). Although some of the Wnt5a effects are independent of Ror2 and may involve other similar kinase (such as Ror 1 or Ryk) (Fukuda *et al*., 2008; Keeble *et al*., 2006), genetic evidences highlight the relevance of Ror2 in Wnt5a signaling, as Ror2 depletion in mice leads to embryonic defects that mirror phenotypes observed in Wnt5a‐null mice (Ho *et al*., 2012). Wnt5a‐induced formation of the complex between Fz and Ror2 precedes Dvl2 phosphorylation and polymerization and other downstream responses (Nishita *et al*., 2010). Different evidences indicate that CK1ε is also involved in noncanonical Wnt signaling being required for Dvl2 phosphorylation (Bryja *et al*., 2007; Klein *et al*., 2006; Strutt *et al*., 2006). As p120‐catenin is needed for CK1ε action in canonical Wnt signaling, we have investigated if CK1ε activation by Wnt5a also requires p120‐catenin. Our results indicate that CK1ε binding to the Fz complex does not involve p120‐catenin as this kinase directly associates with Ror2. CK1ε binding to Ror2 is not just necessary for CK1ε activation but also protects Ror2 protein from degradation. Finally, we demonstrate that p120‐catenin is also relevant in this pathway as it also associates with Ror2 and is necessary to maintain this protein in the plasma membrane.

## 2 Materials and methods

### 2.1 Antibodies

The antibodies used in this study and their specific utilization in different assays are presented in Table S1.

### 2.2 Cell culture

HEK293T, SW‐480, and IEC‐18 cells were obtained from the IMIM Cell Bank and cultured in Dulbecco's modified Eagle's medium (DMEM; Invitrogen, Carlsbad, CA, USA) supplemented with 10% fetal bovine serum (Invitrogen) at 37 °C in 5% CO_2_. Assays were performed with cells at 60–70% confluency. The generation and use of murine mesenchymal stem cells (MSCs) has been reported (Alba‐Castellón *et al*., 2016). Control L fibroblasts or fibroblasts stably transfected with plasmid encoding Wnt3a or Wnt5a were obtained from the ATCC (ref. CRL‐2648, CRL‐2647, and CRL‐2814, respectively). Wnt3a‐L and Wnt5a‐L fibroblasts were cultured in medium containing 0.4 mg·mL^−1^ G‐418. Conditioned medium was collected from the corresponding cells cultured for 4 days without antibiotic. Where indicated, the CK1 inhibitor IC261 (400090; CalbioChem, San Diego, CA, USA) (15 μm) or the Src tyrosine kinases inhibitor herbimycin A (Hb) (H6649; Sigma, St. Louis, MO, USA) (20 ng·mL^−1^) were supplemented to the cell medium.

### 2.3 Cell transfection and selection of transfectants

Human shRNA specific for p120‐catenin (#TRCN122988, #TRCN122987), CK1ε (#TRCN1837, #TRCN1836), PR61ε (#TRCN2558, #TRCN2560), Ror2 (#TRCN1492, #TRCN10625), or a nontargeting control (#SHC001, #SHC002) were obtained from Mission shRNA (Sigma). For transient expression of shRNA in HEK293T cells, the indicated shRNA were transfected using polyethylenimine (PEI) (Polysciences Inc., Worrington, PA, USA) and downregulation of the investigated protein was analyzed at 48–72 h after transfection by Western blot (WB). Transient overexpression of ectopic Ror2‐HA or Ror2‐Flag (kindly provided by Y. Minami, Kobe University, Japan) was achieved by transfecting the indicated eukaryotic plasmid using PEI. Cells were analyzed 24–48 h after transfection.

### 2.4 Generation of HEK293T CK1ε and p120‐catenin KO cells by CRISPR/Cas9

Five different gRNA/Cas9 sequences against CK1ε or p120‐catenin were inserted into pD1301‐AD (Horizon Discovery, Cambridge, UK), a plasmid that also expresses Cas9, and transfected individually in HEK293T cell line using Lipofectamine 2000 and according to manufacturer's instructions. The gRNA sequences used for CK1ε were gRNA1, 5′‐ATGGCTCACTCTTGCTGCAG‐3′; gRNA2, 5′‐TCCCGATCTTCCGTCCCAGG‐3′; gRNA3, 5′‐ACCCAGGTAGATATCTCCGA‐3′; gRNA4, 5′‐GCGATGTTGGCACCTGCCCG‐3′; gRNA5, 5′‐TGAGGAAGTCGCCATCAAGC‐3′; and those for p120‐catenin were gRNA1, 5′‐TAGGTCTCCACAATCTGCCC‐3′; gRNA2, 5′‐ACTACAGACATGGCTCCCTC‐3′; gRNA3, 5′‐CCGAGTGGTCCCATCATCTG‐3′; gRNA4, 5′‐ACACACGTCTTAGTTTACCG‐3′; and gRNA5, 5′‐ACTTTCTTGACCTGCAAGT‐3′. gRNA off‐target analyses were performed without statistical significance. As pD1301‐AD also expresses GFP, 72 h post‐transfection GFP‐positive cells were sorted with FACSAria II SORP (BD Bioscience, San Jose, CA, USA) and individually plated into a 96‐well plate. After cell clones were grown, CK1ε or p120‐catenin protein expression was checked by WB. Clones with no CK1ε or p120‐catenin protein expression were selected.

### 2.5 Purification of recombinant proteins, pull‐down, and phosphorylation

Generation of the bacterial expression plasmid pGEX‐6P encoding the glutathione S‐transferase (GST) fused to p120‐catenin wild‐type or to p120‐catenin deletion fragments has been previously described (Castaño *et al*., 2007; Roura *et al*., 1999). The cytoplasmic domain of Ror2 (amino acids 426–944, cytoRor2) was amplified from pcDNA‐mRor2‐HA, using the primers 5′‐AATAAGGGATCCTGCATGTGCCGCAAC‐3′ (forward) and 5′‐TTAATAGAATTCCATGAGCCGCCTCGG‐3′ (reverse). The PCR product was then digested with BamHI and EcoRI and inserted into pGEX‐6P3 to generate GST–cytoRor2. The deletion Ror2 fragments were produced as follows: cytoRor2 (aa 426–563) was obtained by cleaving pGEX‐6P3‐cytoRor2 with EcoRI and relegating; cytoRor2 (aa 563–944) was obtained by cleaving pGEXP3‐cytoRor2 with EcoRI and inserting the corresponding band into EcoRI‐digested pGEX‐6P1. To prepare GST–CKε fusion protein, full‐length CK1ε cDNA was obtained by PCR from mRNA of the HEK293T cells using the primers 5′‐TAACCCGGGATGGAGCTACGTGTGGGGA‐3′ (forward) and 5′‐TTAGCGGCCGCTCACTTCCCGAGATGGTCA‐3′ (reverse), flanked by XmaI and NotI, respectively. After enzymatic digestion, the PCR product was inserted into pGEX‐6P3.

GST fusion proteins were expressed in *Escherichia coli* and purified by affinity chromatography on glutathione‐Sepharose columns as described (Roura *et al*., 1999). Where indicated, cyto‐Ror2 or p120‐catenin were phosphorylated using 300 milliunits of recombinant catalytic domain of protein kinase CK1 (New England Biolabs, Ipswich, MA, USA) or Src (Millipore, Darmstadt, Germany, Src 1‐530 active) in a final volume of 50 μL, with 50 mm Tris/HCl pH 7.5, 10 mm MgCl_2_, 0.1 mm EDTA, 2 mm DTT, 2.5 mm β‐glycerol phosphate (only for CK1 phosphorylation) pH 7.0, and 100 μm ATP. Reactions were performed for 30 min at 30 °C. Where required, GST was removed using Pre‐Scission protease (Amersham Biosciences, Waltham, MA, USA) as reported (Castaño *et al*., 2007). Pull‐down assays were performed using recombinant GST protein as bait together with purified protein or HEK293T cell extracts (500–700 μg) that had been lysed with NP‐40 0.5% lysis buffer. Binding assays were performed at 4 °C for 2 h, after which 20 μL of glutathione‐Sepharose beads was added at 4 °C for 1 h more. After washing, pull‐down proteins were analyzed by WB with specific antibodies (Table S1).

### 2.6 Rac activity

Rac1 activity was determined in HEK293T cells using specific pull‐down assays for the activated form of this protein. Active Rac1 was affinity‐precipitated using the Rac1 binding domain of PAK as described (Valls *et al*., 2012).

### 2.7 Immunoprecipitation

Cell extracts were prepared by homogenizing cells in 0.5% NP‐40 lysis buffer (25 mm Tris/HCl pH 7.6, 150 mm NaCl, 1 mm EDTA, 0.5% NP‐40), supplemented with protease and phosphatase inhibitors as described (Casagolda *et al*., 2010). Extracts were left on ice for 10 min and centrifuged at 14 000 ***g*** for 10 min at 4 °C. Supernatants constituted the cell extracts. Proteins were immunoprecipitated from cell extracts (300–600 μg) using 1 μg·mL^−1^ of the appropriate antibody, or an irrelevant IgG as a control, for 16 h at 4 °C. Samples were incubated for 2 h with 20 μL of γ‐bind G‐Sepharose (GE Healthcare, Pittsburg, PA, USA). Immunoprecipitates were washed 3× with PBS‐0.1% NP‐40, and bound proteins were analyzed by WB.

### 2.8 CK1ε activity

Protein was immunoprecipitated from HEK293T total cell extracts with CK1ε mAb for 4 h at 4 °C. Immunoprecipitates were washed 3× with 0.1% NP‐40 lysis buffer and once with phosphorylation buffer (50 mm Tris/HCl pH 7.5, 10 mm MgCl_2_, 0.1 mm EDTA, 2 mm DTT, 2.5 mm β‐glycerol phosphate). The immunocomplexes were then incubated with recombinant GST–p120‐catenin and phosphorylation buffer supplemented with 100 μm ATP in a final volume of 50 μL for 30 min at 30 °C. Specific phosphorylation on GST–p120‐catenin Ser268 was analyzed by WB with a phospho‐specific Ser268‐p120‐catenin monoclonal antibody (Table S1).

### 2.9 RNA isolation and analysis

RNA were obtained as previously reported (Solanas *et al*., 2008) and analyzed by semi‐quantitative reverse transcription polymerase chain reaction (RT‐PCR) in triplicate. The primers used were as follows: *ROR2*, forward: 5′‐ATCGCCCGCTCCAACCCTCTCATC‐3′, and reverse: 5′‐ATCCCCATCTTGCTGCTGTCTCG‐3′; *SIAH2*, forward: 5′‐GCTAATAAACCCTGCAG A‐3′, and reverse: 5′‐ACTTCTGGCGGCATTGGTCATA‐3′, and *GAPDH*, forward: 5′‐ACCACAGTCCATGCCATCAC‐3′, and reverse: 5′‐TCCACCACCCTGTTGCTGTA‐3′.

### 2.10 Luciferase reporter

Cells were transfected as above with the TOP‐Flash plasmid, a synthetic promoter sensitive to the activity of the β‐catenin–Tcf‐4 complex that contains three copies of the Tcf‐4 binding site upstream of a firefly luciferase reporter gene. A mutated version of this plasmid (FOP‐Flash) was used as control. Activity of the product of the Renilla luciferase gene under the control of a constitutive thymidine kinase promoter (Promega, Madison, WI, USA) was used to normalize transfection efficiency.

### 2.11 Collagen type I invasion

4–7 × 10^4^ cells were suspended in 150 μL DMEM 0.1% BSA, were seeded on a Transwell filter chamber (Costar 3422; Thermo Fisher Scientific, Waltham, MA, USA) coated with 1 mg·mL^−1^ collagen type I (354249; Corning, Corning, NY, USA) and incubated for 16 h (MSC) or 36 h (HEK293T). Control or Wnt5a‐conditioned medium was used as chemoattractant. Noninvading cells were removed from the upper surface of the membrane, while cells that adhered to the lower surface were fixed with Methanol 100% for 20 min and stained with crystal violet. Cells were eluted with 30% acetic acid, and the OD was measured at 590 nm.

### 2.12 Single‐cell cortical actin polarization analysis

About 40 μL of Matrigel (354230; BD Biosciences) was deposited on a round coverslip (15 mm diameter) and incubated at 37 °C for 30 min to solidify the gel. Cells were trypsinized to a single‐cell suspension, and 5 × 10^4^ cells in control or Wnt5a‐conditioned medium and 2% Matrigel (V/V) were seeded on top of the solidified gel. Cells were incubated for 2 h with the indicated media and analyzed by immunofluorescence. For immunofluorescence, cells were first fixed with 4% paraformaldehyde for 15 min and incubated with PBS‐0.2% Triton X‐100 for 5 min. After blocking with 3% BSA in PBS for 30 min at room temperature, CytoPainter Phalloidin‐iFluor 647 Reagent (176759; Abcam, Cambridge, UK) was added for 1 h at room temperature. Slides were washed three times with PBS and incubated for 10 min with 4′,6‐diamidino‐2‐phenylindole (Dapi) for nucleus identification. Coverslips were mounted on glass slides with Fluoprep (75521; BioMérieux, Marcy l'Etoile, France), and immunofluorescence was analyzed with a Leica confocal microscope (LEICAspectral confocal TCS‐SL, Leica, Buffalo Grove, IL, USA).

### 2.13 Cell surface protein digestion with Proteinase K

Cells were treated with Proteinase K (Invitrogen) at 1 μg·mL^−1^ for 10 min at room temperature. Digestion was stopped with 4‐(2‐aminoethyl)benzenesulfonyl fluoride hydrochloride (20 mm), and cells were lysed with RIPA lysis buffer (25 mm Tris pH 7.6, 210 mm NaCl, 1 mm EGTA, 1% Nonidet‐40, 0.5% sodium deoxycholate, 0.1% SDS) supplemented with protease inhibitors (Casagolda *et al*., 2010) and heated at 60 °C for 5 min.

### 2.14 Cell surface biotinylation

Cells were incubated with 0.5 mg·mL^−1^ sulfo‐NHS‐LC‐biotin (Pierce, Rockford, IL, USA) at 4 °C for 30 min. After quenching excess of biotin with 50 mm NH_4_Cl, cells were lysed in 0.5% NP‐40 lysis buffer as described above. Lysates were incubated with NeutrAvidin Agarose (Sigma). Beads were collected, washed 3× with PBS‐0.1% NP‐40 buffer, and analyzed by WB.

### 2.15 Statistical analysis

Results are representative of at least three independent experiments unless otherwise indicated. Data are presented as mean ± SD. Where appropriate, statistical analyses were conducted using graphpad prism software (GraphPad, La Jolla, CA, USA), and data were analyzed for significance using unpaired Student's *t*‐test. *P <* *0.05; **P <* *0.01.

## 3 Results

### 3.1 Canonical and noncanonical Wnt ligands stimulate Dvl2 recruitment to Fz

**Figure 1:**
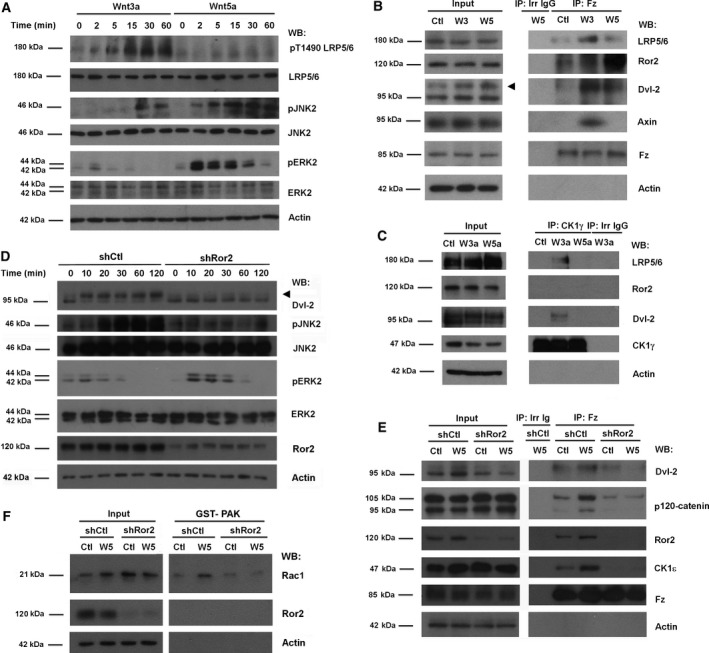
Noncanonical Wnt promotes Ror2‐dependent Dvl2 binding to Fz. (A) HEK293T cells were stimulated with Wnt3a‐ or Wnt5a‐conditioned medium for the indicated times. Cells were lysed, and proteins were analyzed by WB with specific antibodies. JNK2, ERK2, and LRP5/6 phosphorylation were determined with anti‐phospho antibodies against JNK (Thr183/Tyr185, Thr221/Tyr223), ERK (Thr202/Tyr2014), or LRP5/6 (Thr1490). (B, C) HEK293T cells treated with control, Wnt3a‐ or Wnt5a‐conditioned medium for 15 min were lysed, and total Fz (B) or CK1γ (C) were immunoprecipitated with specific antibodies. Associated proteins were analyzed by WB. (D) HEK293T cells depleted of Ror2 with specific shRNA were stimulated with control or Wnt5a‐conditioned medium for the indicated times. Cells were lysed, and Dvl2 phosphorylation was determined by the shift in the molecular weight of Dvl2, and JNK2 and ERK2 phosphorylation as above. The arrow indicates phosphorylated Dvl2. (E, F) HEK293T cells depleted of Ror2 were treated with control or Wnt5a‐conditioned medium for 5 min (E) or 1 h (F). In (F), a GST–PAK pull‐down assay was performed, and active Rac1 was determined by WB. In (E), Fz was immunoprecipitated from total extracts, and associated proteins were analyzed by WB.

Dvl‐2 phosphorylation and binding to Fz is a common response to Wnt ligands acting through both the canonical and noncanonical pathways (González‐Sancho *et al*., 2004). We confirmed these results in the widely used system of HEK293T cells. Wnt3a that stimulates the canonical signaling (hereby referred to as ‘canonical Wnt’) and Wnt5a that acts through the noncanonical one (‘noncanonical Wnt’) elicited differential responses, with only Wnt3a stimulated the phosphorylation of Thr1490 in LRP5/6 co‐receptor (Fig. 1A). In contrast, Wnt5a and not Wnt3a rapidly and transiently stimulated ERK2 (Prasad *et al*., 2013). Also as reported (Wu *et al*., 2008; Yamanaka *et al*., 2002), both factors increased JNK2 activity, although Wnt5a did it faster and more potently. Dvl2 phosphorylation, detected by a shift in electrophoretic mobility, was also augmented after stimulation by both Wnt3a and Wnt5a (Fig. 1B). These two factors also promoted Dvl2 association with Fz detected by co‐immunoprecipitation experiments (Fig. 1B). The specificity of the Wnt5a effects was confirmed using a Wnt5a‐blocking antibody that prevented the effects of this factor on ERK2 and JNK2 phosphorylation (Fig. S1A), as well as the Wnt5a‐induced Fz–Dvl2 interaction (Fig. S1B). Other canonical Wnt responses, such as CK1γ association with LRP5/6 and Dvl2, were only observed upon Wnt3a addition to the cells (Fig. 1C). Wnt3a and Wnt5a induced the interaction of Fz with their specific co‐receptors: Wnt3a but not Wnt5a increased the Fz association with LRP5/6 (and binding to Axin) (Fig. 1B), whereas only Wnt5a promoted the assembly of the Ror2–Fz complex (Fig. 1B). These results demonstrate that Wnt5a responses are specific and do not activate the canonical pathway.

We next examined the relevance of Ror2 for the Wnt5a responses. Corroborating previous results (Ho *et al*., 2012), we found that Ror2 down‐modulation by a specific shRNA blocked the rapid Wnt5a‐induced Dvl2 phosphorylation (Fig. 1D) as well as Dvl2 binding to Fz (Fig. 1E). Other later responses to Wnt5a were also prevented, such as Rac1 activation (Fig. 1F) and JNK2 phosphorylation (Fig. 1D), but the transient activation of ERK2 was not affected (Fig. 1D), indicating that this Wnt5a effect is not mediated by Ror2. The tyrosine kinase inhibitor herbimycin (Fukazawa *et al*., 1991) completely blocked Ror2 tyrosine phosphorylation (Fig S2A) and inhibited the Wnt5‐induced Dvl2 association with Fz and Rac1 activation (Fig. S2B‐C). These results confirm previous reports (see Section 1) indicating that, although Wnt5a acts through a different co‐receptor than Wnt3a, it also promotes the association of Dvl2 with Fz.

### 3.2 p120‐catenin and CK1ε are required for Dvl2 phosphorylation after noncanonical Wnt stimulation

**Figure 2:**
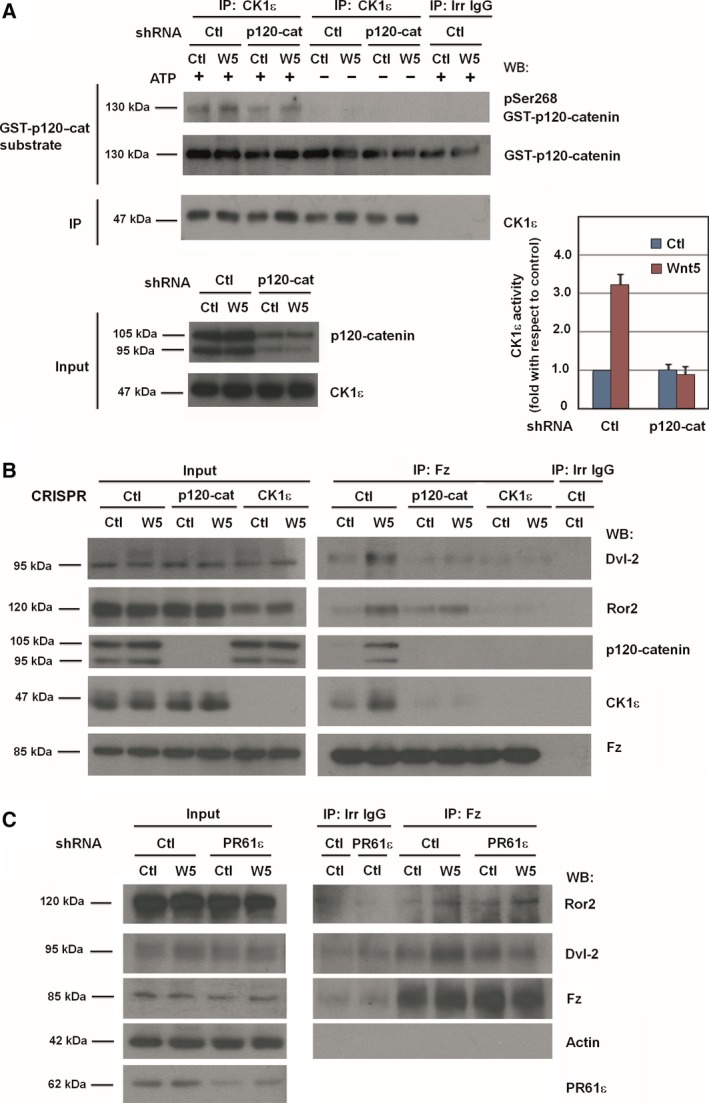
p120‐catenin, CK1ε, and PR61ε are necessary for the activation of noncanonical Wnt pathway. (A) Control or p120‐catenin‐depleted HEK293T cells were treated with control or Wnt5a‐conditioned medium for 15 min. CK1ε was immunoprecipitated from total cell extracts, and the immunocomplex was incubated with 2 pmol of recombinant GST–p120‐catenin in CK1 phosphorylation conditions. Phosphorylation of Ser268 in GST–p120‐catenin was analyzed by WB with a specific PSer268 p120‐catenin antibody. Signal was densitometered, normalized with respect to the GST–p120‐catenin, and represented. The quantification of three different experiments is shown (mean ± SD). The extent of p120‐catenin downregulation by the shRNA is shown in the bottom panel. (B) Fz2 was immunoprecipitated from control, p120‐catenin, or CK1ε HEK293T CRISPR whole‐cell extracts treated with control or Wnt5a‐conditioned medium for 5 min. Protein complexes were analyzed by WB with the indicated antibodies. (C) HEK293T cells depleted of PR61ε using specific shRNA, or a scrambled shRNA as control, were treated with control or Wnt5a‐conditioned medium for 5 min. Fz2 was immunoprecipitated from total cell extracts, and the protein complex was analyzed by WB.

CK1ε participates in Dvl2 phosphorylation by canonical (Cong *et al*., 2004; Swiatek *et al*., 2004) and noncanonical Wnts (Bryja *et al*., 2007). Activation of CK1ε by Wnt3a requires its interaction with p120‐catenin (Casagolda *et al*., 2010). Therefore, we investigated whether the CK1ε–p120‐catenin complex is also involved in Dvl2 recruitment in noncanonical Wnt signaling. First, we observed that Wnt5a increased the association of both p120‐catenin and CK1ε with Fz (Fig. 1E, see also Fig. 2B). We thus tested the activity of CK1ε from control cells or Wnt5a‐stimulated cells. For that, CK1ε was immunoprecipitated and its activity assayed *in vitro*, using recombinant GST–p120‐catenin as a substrate. Notably, phosphorylation of p120‐catenin increased if incubated with CK1ε from Wnt5a‐treated cells as compared to that from control cells, even though similar levels of CK1ε were present in the immunocomplexes in both cases (Fig. 2A). No substrate phosphorylation was observed when ATP was omitted from the reaction. The increase in GST‐p120‐catenin phosphorylation was calculated to be approximately threefold, as determined from three different experiments (Fig. 2A). Thus, CK1ε intrinsic activity was stimulated by Wnt5a. Further, p120‐catenin downregulation by a shRNA prevented the Wnt5a‐induced activation of CK1ε (Fig. 2A).

We next investigated the role of p120‐catenin and CK1ε in other reactions induced by Wnt5a. For this, and to validate the results obtained with shRNA, we generated HEK mutants lacking p120‐catenin or CK1ε using the CRISPR/Cas9 technology. These cells do not express the knocked‐down proteins (Fig. 2B). Importantly, CK1ε‐depleted cells did not show any alterations in CK1δ, the CK1 family member most similar to CK1ε (Fig. S3). p120‐catenin or CK1ε depletion affected Wnt5a‐induced Dvl2 interaction with Fz, as the amount of Dvl2 co‐immunoprecipitated with Fz decreased in cells depleted of either of these elements (Fig. 2B). This result suggests that p120‐catenin, alike CK1ε, is required for assembling the Wnt5a‐signalosome, as it is for the Wnt3a‐induced complex (Del Valle‐Pérez *et al*., 2011; Swiatek *et al*., 2004; Vinyoles *et al*., 2014). However, our results show significant differences between the two pathways; for instance, whereas CK1ε depletion did not modify LRP5/6 levels (Casagolda *et al*., 2010; see also Fig. S11A), it decreased total Ror2 protein (Fig. 2B). Moreover, p120‐catenin depletion did not alter total Ror2 (Fig. 2B) indicating a significant difference between p120‐catenin and CK1ε elimination in this pathway.

In canonical Wnt signaling, CK1ε is activated by PP2A phosphatase. The action of this phosphatase on CK1ε is mediated by the PR61ε regulatory subunit that interacts with the cytosolic C‐tail of Fz (Vinyoles *et al*., 2017). Correspondingly, PR61ε downregulation prevented the Wnt5a‐induced increase in the amount of Dvl2 co‐immunoprecipitated with Fz (Fig. 2C). On the other hand, it did not alter the levels of Ror2 present in the Fz immunocomplex (Fig. 2C), indicating that PR61ε is not required for the formation of the Fz–Ror2 complex. Therefore, these results show that, similar to canonical Wnt signaling, CK1ε is also activated by Wnt5a through the action of the PR61ε–PP2A complexes and is required for Dvl2 recruitment to Fz receptor.

Other Wnt5a responses also showed a requirement for p120‐catenin and CK1ε. In addition to interfering with Dvl2 phosphorylation, depletion of p120‐catenin precluded JNK2 phosphorylation and Rac1 activation (Fig. S4A, B). However, it did not block ERK2 activation; on the contrary, it enhanced this phosphorylation, suggesting that JNK2 and ERK2 belong to different branches of the noncanonical Wnt pathway. In line with this, JNK2 and ERK2 activation showed a different sensitivity to Ror2 depletion (see Fig. 1D). Cells with abrogated CK1ε expression also showed an impaired JNK2 phosphorylation and Rac1 activation in response to Wnt5a (Fig. S5A, B).

### 3.3 p120‐catenin interacts with Ror2 and controls Ror2 endocytosis

As our results indicated that both p120‐catenin and CK1ε are required for noncanonical Wnt signaling, we next examined if these two proteins participate in the pathway forming a complex. p120‐catenin and CK1ε associated with Fz when cells were stimulated with Wnt5a (see Figs 1E and 2B), similar to what occurs with Wnt3a. Binding of p120‐catenin to Fz is not direct; in Wnt3a‐stimulated cells, it is dependent on N‐cadherin and LRP5/6 (Casagolda *et al*., 2010). However, neither LRP5/6 (see Fig. 1B) nor N‐cadherin (Fig. S6A) were recruited to Fz upon Wnt5a stimulation. Likewise, Ror2 did not interact with N‐cadherin (Fig. S6B). N‐cadherin down‐modulation did not prevent Wnt5a‐induced Fz interactions with Dvl2, p120‐catenin, or CK1ε (Fig. S6A), ruling out that the p120‐catenin and CK1ε interactions with Ror2 are mediated by N‐cadherin. Thus, we investigated the molecular basis of Fz–p120‐catenin association in noncanonical Wnt pathway.

**Figure 3:**
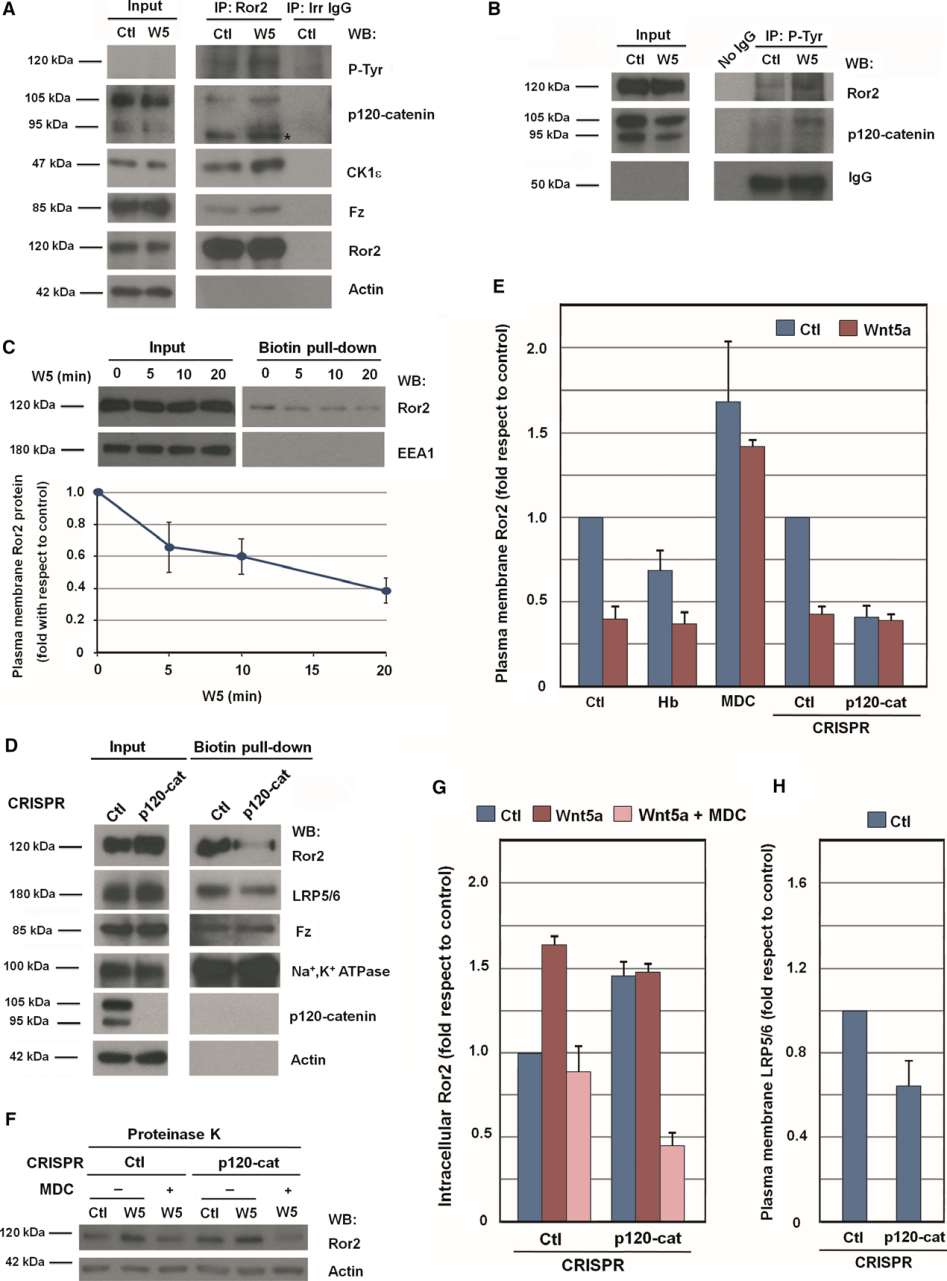
Ror2 internalization is controlled by p120‐catenin. HEK293T cells were treated with control or Wnt5a‐conditioned medium for 5 min. Cells were lysed, and Ror2 (A) or tyrosine‐phosphorylated proteins (B) were immunoprecipitated with specific antibodies. Associated proteins were analyzed by WB. The asterisk in (A) indicates an unspecific band. (C, D) Surface proteins were biotinylated in HEK293T cells treated with control or Wnt5a‐conditioned medium for the indicated times (C), or in control and p120‐catenin HEK293T CRISPR cells (D). A pull‐down assay was performed with NeutrAvidin Agarose, and biotinylated membrane proteins were analyzed by WB. In (C, bottom), Ror2 protein levels at plasma membrane were quantified by analyzing three independent experiments (mean ± SD). (E) Control or p120‐catenin HEK293T CRISPR cells were pretreated with 20 ng·mL ^−1^ herbimycin (Hb) for 1 h, or with 50 μm monodansylcadaverine (MDC) for 30 min, as indicated. Cells were then stimulated with control or Wnt5a‐conditioned medium for an additional 20 min. After cell surface biotinylation, lysates were precipitated with NeutrAvidin Agarose. The amount of cell surface Ror2 was quantified and represented. (F) Control or p120‐catenin HEK293T CRISPR cells were pretreated with 50 μm MDC for 30 min. Cells were then stimulated with control or Wnt5a‐conditioned medium for an additional 20 min with MDC, as indicated. Intact cells were treated with proteinase K for 10 min. Total cell extracts were prepared, and total Ror2 levels were analyzed by WB. (G, H) Autoradiograms from the three different experiments performed in (D) and (F) were quantified and represented. In (G), intracellular Ror2 levels were quantified, and in (H), LRP5/6 plasma membrane levels were quantified.

p120‐catenin interacts with Ror2, as observed by co‐immunoprecipitation experiments (Fig. 3A and Fig. S7). Formation of the complex was detected in nontreated cells and was significantly stimulated by Wnt5a. Ror2 association with p120‐catenin was blocked by the Tyr kinase inhibitor Hb, indicating that it requires tyrosine phosphorylation (Fig. S7). Both p120‐catenin and Ror2 were phosphorylated in tyrosine residues under basal conditions, as observed by immunoprecipitation and Western blot experiments using anti‐Ror2, anti‐p120‐catenin, and anti‐PTyr antibodies (Fig. 3A, B, Figs S2A and S7); this modification was enhanced by Wnt5a.

As p120‐catenin directly binds several protein tyrosine kinases, such as Fer and Fyn (Kim and Wong, 1995; Piedra *et al*., 2003), we next examined whether p120‐catenin also directly associates with Ror2. Recombinant proteins corresponding to p120‐catenin and the cytosolic domain of Ror2 (cyto‐Ror2) were prepared to analyze the interactions between these two proteins (Fig. S8A). GST–p120‐catenin fusion protein precipitated Ror2 from cells extracts (Fig. S8B). Deletion of the first 102 amino acids (aa) of p120‐catenin did not affect Ror2 binding; however, removal of the entire regulatory domain (aa 1–350) prevented it. These results thus suggest that binding of Ror2 takes place through aa 102–350. As this p120‐catenin domain is phosphorylated by CK1 and Src (Kourtidis *et al*., 2013), we determined the effect of p120‐catenin phosphorylation on its interaction with Ror2. Notably, p120‐catenin phosphorylation by Src, but not by CK1, increased the p120‐catenin/Ror2 interaction (Fig. S8C).

Pull‐down assays were repeated using the two recombinant proteins. A GST–cytoRor2 fusion protein bound recombinant p120‐catenin (Fig. S8D). The association required the juxtamembrane sequence of Ror2, as it was also observed with a GST fusion protein comprising aa 426–563 of Ror2. In contrast, the remaining Ror2 C‐terminal domain (aa 563–944) did not significantly interact with p120‐catenin. Although GST–cytoRor2 was efficiently phosphorylated by Src, this modification did not enhance its binding to p120‐catenin (Fig. S8E).

Wnt5a promotes clathrin‐dependent Fz‐receptor complex internalization (Sato *et al*., 2010; Shojima *et al*., 2015). These results were reproduced in our cellular system analyzing biotinylation of membrane proteins in intact cells. Wnt5a caused a progressive decrease in biotinylated Ror2, suggesting internalization of this protein (Fig. 3C). In contrast, an internal membrane protein used as a control, EEA1, was not biotinylated. As previously reported (Sato *et al*., 2010), addition of monodansylcadaverine (MDC), an inhibitor of clathrin‐dependent endocytosis, prevented the disappearance of Ror2 from the plasma membrane (Fig. 3E, F and Fig. S9A).

We have shown that p120‐catenin is tyrosine phosphorylated in response to Wnt5a increasing its interaction with Ror2 (see Fig. 3A and Fig. S8C). Moreover, the tyrosine kinase inhibitor Hb decreased Ror2 levels in the plasma membrane (Fig. 3E and Fig. S9B) and blocked other responses to Wnt5a, either initial, such as Dvl‐2 association with Fz (see Fig. S2A) or late, such as Rac1 and JNK activation (Fig. S9B, see also Fig. S2B).

We reasoned that p120‐catenin might control Ror2 internalization, acting in a similar fashion to its function on E‐cadherin (Kourtidis *et al*., 2013). Elimination of p120‐catenin in CRISPR cells decreased the levels of Ror2 in the plasma membrane, as detected by biotinylation experiments (Fig. 3D, E). In p120‐catenin‐depleted cells, presence of Ror2 in the membrane was decreased by 60% (Fig. 3E). The same conclusion was obtained in experiments in which cells were treated with proteinase K: the levels of protease‐resistant (intracellular) Ror2 increased following Wnt5a stimulation in a MDC‐sensitive fashion (Fig. 3F, G). Intracellular Ror2 was also upregulated in p120‐catenin knockout (KO) cells. This increase was prevented in cells treated with MDC, further demonstrating the role of p120‐catenin in controlling clathrin‐dependent internalization of Ror2. The presence in the plasma membrane of LRP5/6, the co‐receptor of the canonical Wnt signaling pathway, was also diminished by elimination of p120‐catenin (Fig. 3D), although to a lower extent (Fig. 3H).

### 3.4 Ror2 directly associates with CK1ε and prevents its lysosomal degradation

**Figure 4:**
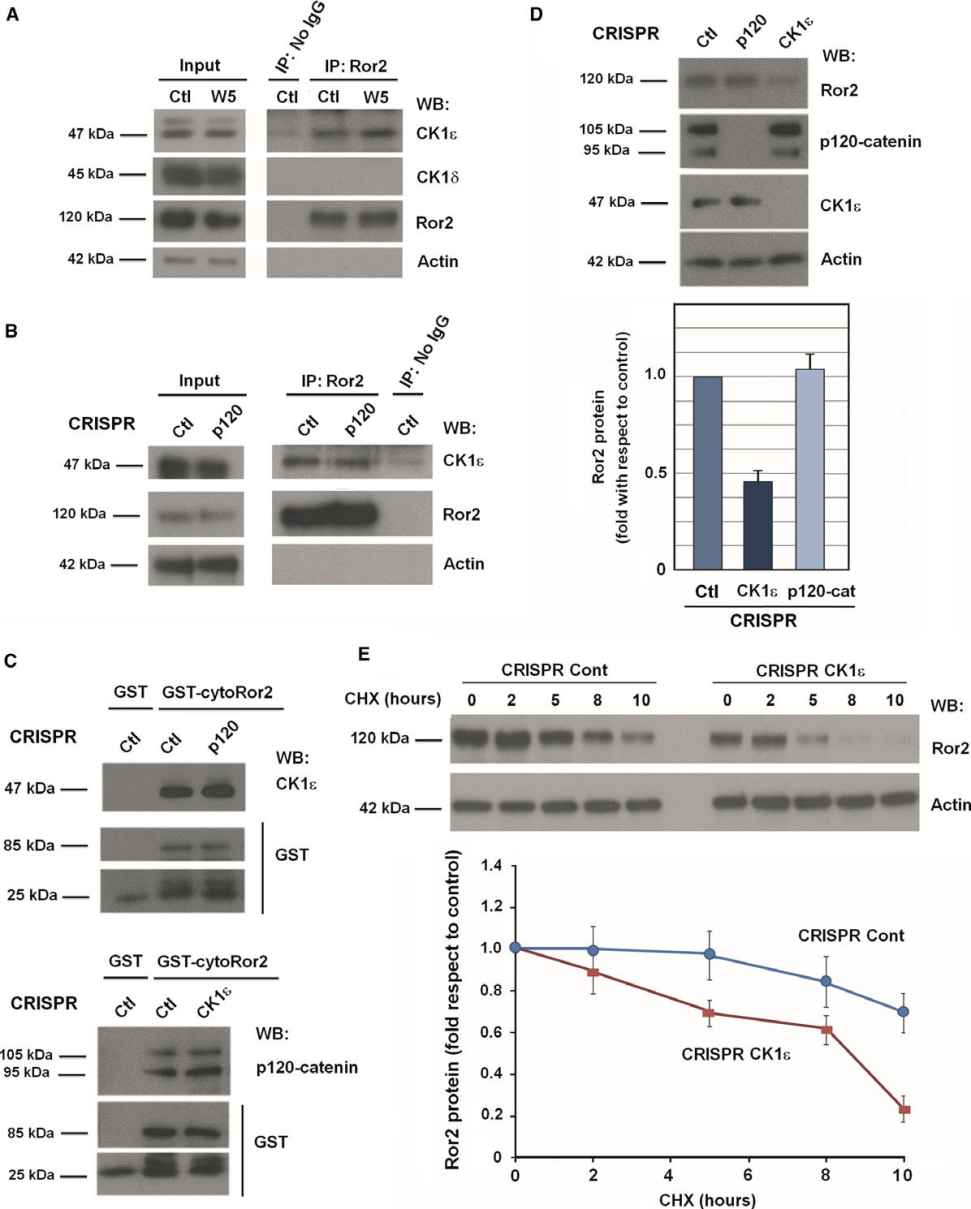
CK1ε binds and stabilizes Ror2. (A, B) Control or p120‐catenin HEK293T CRISPR cells were treated with control or Wnt5a‐conditioned medium for 5 min, and Ror2 was immunoprecipitated from total cell extracts. Associated proteins were analyzed by WB. (C) Pull‐down assays were performed by incubating 700 μg total cell extracts from control, p120‐catenin, or CK1ε HEK293T CRISPR cells with 10 pmol of GST–cyto‐Ror2. Protein complexes were affinity purified and analyzed by WB. (D) Control, p120‐catenin, and CK1ε HEK293T CRISPR cells were lysed and analyzed by WB (top). Autoradiograms from three different experiments were quantified, and total Ror2 protein levels are shown (mean ± SD) (bottom). (E) Control or CK1ε HEK293T CRISPR were treated with 50 μg·mL ^−1^ cycloheximide for the indicated times. Cells were lysed, and Ror2 protein levels were analyzed by WB (top). Autoradiograms from four different experiments performed were quantified using quantity one software (Bio‐Rad, Hercules, CA, USA) and represented for each time point with respect to the control (mean ± SD) (bottom).

We also analyzed the interaction of CK1ε with Ror2 by co‐immunoprecipitation and found that CK1ε (but not CK1δ) associated with Ror2 (Fig. 4A); this interaction was slightly stimulated by the presence of Wnt5a. Surprisingly, although p120‐catenin binds CK1ε (Casagolda *et al*., 2010) as well as Ror2 (see Fig. 3A and Fig. S8), it was not required for the CK1ε association with Ror2, as these two proteins co‐immunoprecipitated even in the absence of p120‐catenin (Fig. 4B). Indeed, pull‐down assays performed using GST–cytoRor2 as bait confirmed that Ror2 binds CK1ε and p120‐catenin independently; namely, depletion of p120‐catenin did not affect the amount of CK1ε precipitated by GST–Ror2, while elimination of CK1ε did not prevent p120‐catenin–Ror2 binding (Fig. 4C). Finally, the CK1ε binding site was mapped to the C‐terminal domain of Ror2, to aa 563–944 (Fig. S10), in contrast to p120‐catenin, which interacts through aa 426–563 (see Fig. S8D).

As previously shown in Fig. 2B and also in Fig. 4D and Fig. S11A–C, CK1ε KO cells presented lower levels of Ror2. This downregulation was not observed in other Wnt co‐receptors, such as LRP5/6 (Fig. S11A). Treatment with IC261, a potent but not too selective CK1 inhibitor (Cheong *et al*., 2011; Mashhoon *et al*., 2000), did not decrease Ror2 protein (Fig. S11B), suggesting that the CK1ε protein *per se*, rather than its activity, controls Ror2 protein levels. Both endogenous and transfected Ror2 were less expressed in CK1ε CRISPR cells (Fig. S11C). No differences in Ror2 mRNA were observed between CK1ε‐KO cells and control cells (Fig. S11D), suggesting that Ror2 downregulation was due to a lower stability of Ror2 protein in these cells. Indeed, stability assays in cells treated with cycloheximide indicated that the Ror2 protein half‐life decreased in CK1ε‐depleted cells (Fig. 4E), thus demonstrating that Ror2 interaction with CK1ε controls its protein stability. Levels of Ror2 recovered in CK1ε KO cells after addition of chloroquine but not after that of the proteasomal inhibitor MG (Fig. S11E), indicating that the CK1ε interaction protects Ror2 from lysosomal degradation.

### 3.5 Alike CK1ε, p120‐catenin is required for activation of downstream reactions in the Wnt5a pathway

**Figure 5:**
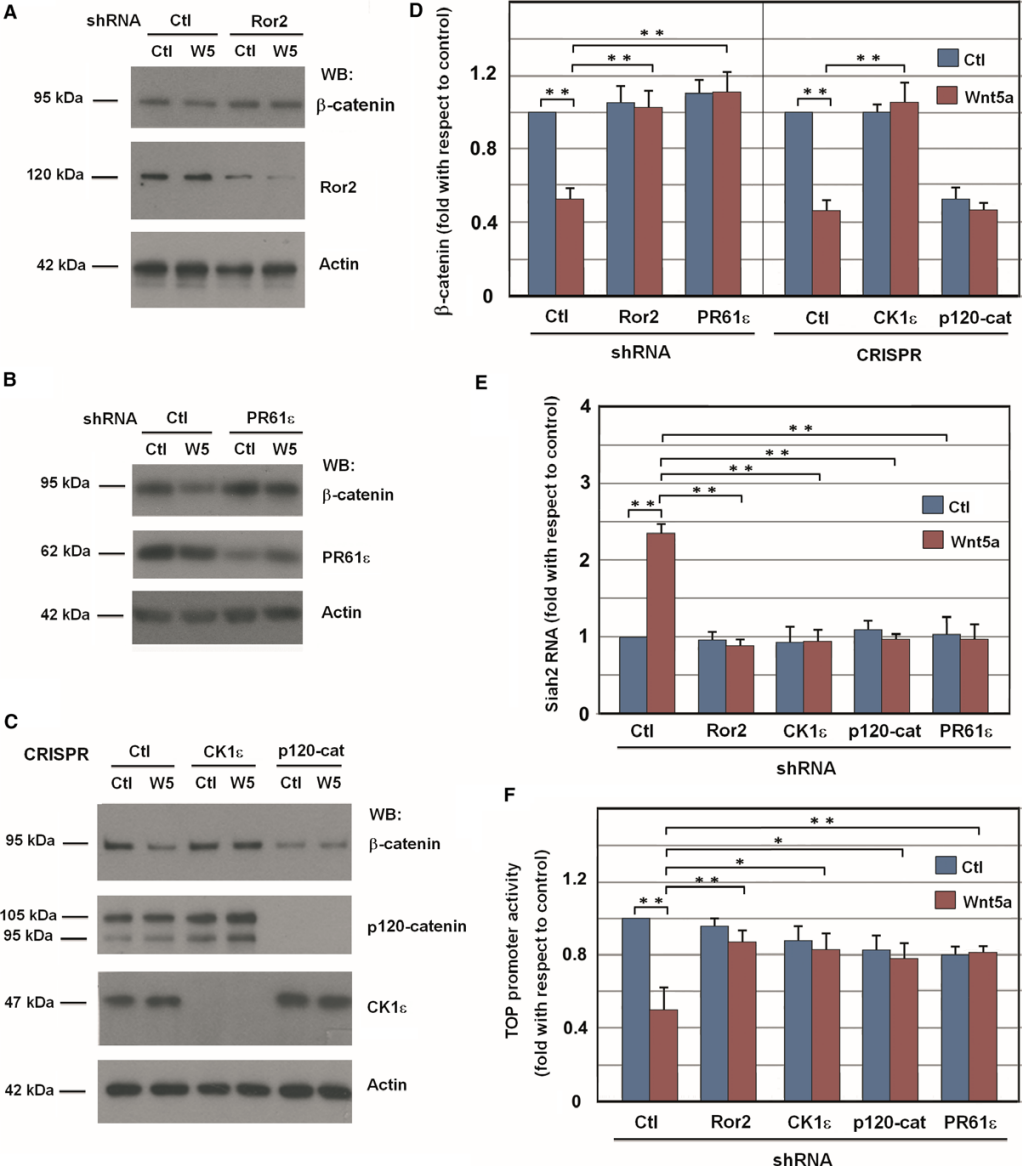
Downregulation of Ror2, p120‐catenin, CK1ε, or PR61ε prevents the β‐catenin down‐modulation induced by Wnt5a. HEK293T cells were depleted of Ror2 (A) or PR61ε (B) using specific shRNA; a scrambled shRNA was used as a control. After 48 h, cells were stimulated with control or Wnt5a‐conditioned medium overnight, and β‐catenin levels were analyzed by WB from total cell extracts. (C) Control, p120‐catenin, and CK1ε CRISPR HEK293T cells were treated with control or Wnt5a‐conditioned medium overnight, and β‐catenin was analyzed by WB. (D) β‐catenin levels were quantified by analyzing three independent experiments performed in (A–C) (mean ± SD). **P <* *0.01. (E) RNA was isolated from control, Ror2, CK1ε, p120‐catenin, and PR61ε‐depleted HEK293T cells stimulated overnight with control or Wnt5a‐conditioned medium. Expression of *SIAH2* was assessed by semi‐quantitative RT‐PCR. Results are presented as mean ± SD from three independent experiments. *P <* *0.05; **P <* *0.01. (F) β‐catenin transcriptional activity was determined using the TOP‐Flash reporter plasmid in SW‐480 cells transfected with the indicated shRNA. pTK‐Renilla plasmid was transfected to normalize the efficiency of transfection. Relative luciferase activity was determined 48 h after transfection. Cells were treated with Wnt5a for 16 h before cell lysis. FOP‐Flash plasmid was also transfected as a control; the activity of this promoter was always lower than 1% of the value of obtained with TOP‐Flash. The mean ± SD of four experiments is shown. **P <* *0.01.

Finally, we determined whether other later responses to Wnt5a stimulation show the same requirement for Ror2, p120‐catenin, and CK1ε. Wnt5a promotes β‐catenin downregulation in a manner that is dependent on transcription of the β‐catenin E3 ligase *Siah2* (Topol *et al*., 2003). This Wnt5a‐induced β‐catenin down‐modulation was not observed in cells depleted of Ror2, CK1ε, or PR61ε (Fig. 5A–D). p120‐catenin depletion decreased β‐catenin levels even under control conditions (Fig. 5C, D). *Siah2* RNA was upregulated by Wnt5a (Fig. 5E) and *Siah2* siRNA prevented the β‐catenin down‐modulation induced by Wnt5a (Fig. S12), indicating the relevance of *Siah2* upregulation for the Wnt5a‐induced β‐catenin decrease. In accordance with these and the previous results, Wnt5a failed to increase *Siah2* RNA in cells deficient for Ror2, CK1ε, p120‐catenin, or PR61ε (Fig. 5E). We also determined whether these proteins were also required for the effects of Wnt5a on β‐catenin‐dependent transcriptional activity, assessed with the widely used TOP promoter. SW‐480 cells were used for these assays as these cells display higher constitutive activity of this promoter. As shown in Fig. 5F, Wnt5a‐induced downregulation of TOP activity was prevented by CK1ε, p120‐catenin, Ror2, or PR61ε shRNA (Fig 5F).

**Figure 6:**
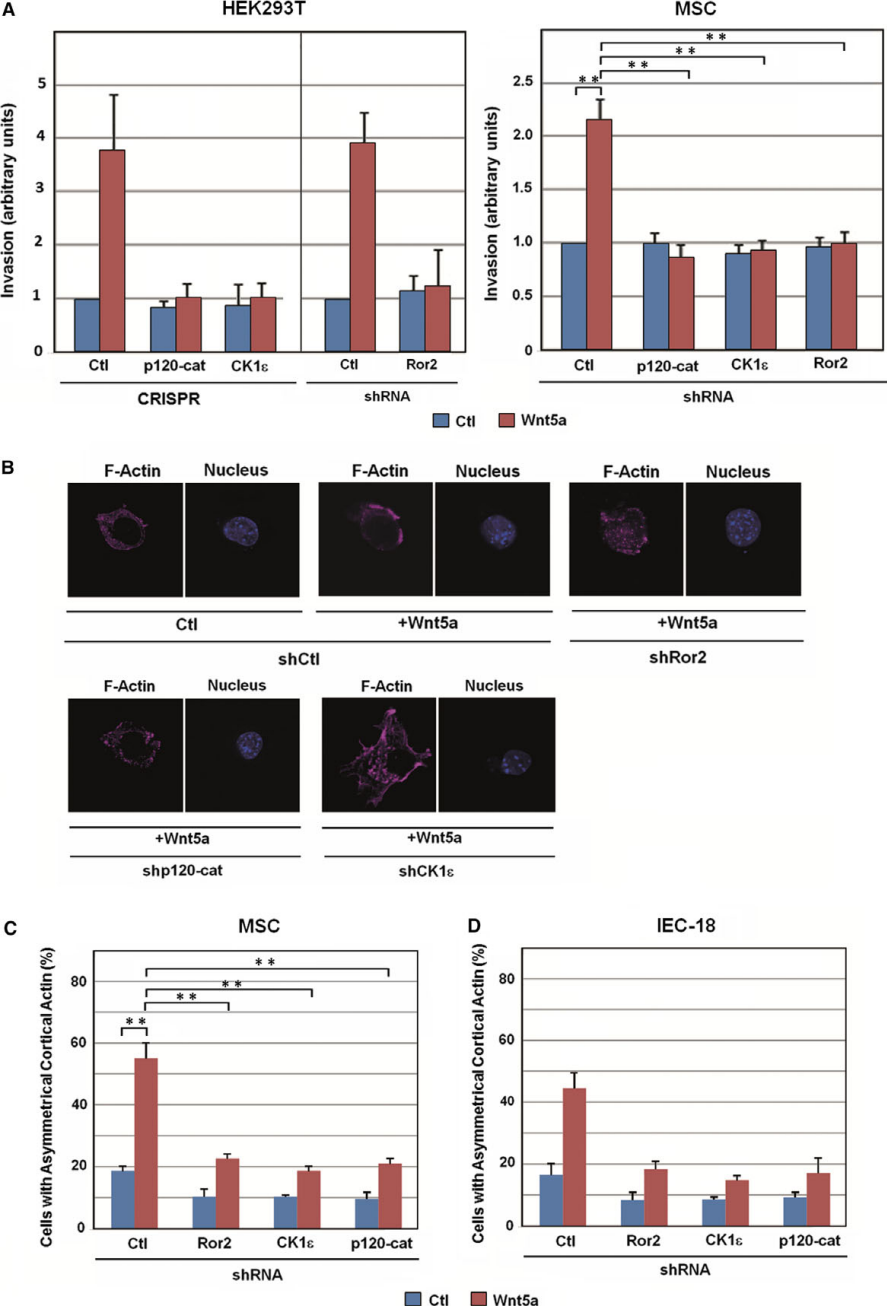
The co‐receptor Ror2, p120‐catenin, and CK1ε are necessary for cell invasion and cortical actin polarization induced by noncanonical Wnt. (A) HEK293T (left) or MSC cells (right) were seeded in Transwell chambers containing 1 mg·mL ^−1^ collagen type I. CRISPR cells or cells transfected with the indicated shRNA were used with control or Wnt5a‐conditioned medium added to the lower chamber. After 16 h (MSC) or 36 h (HEK293) of incubation, cells were fixed and stained with crystal violet, and optical density was quantified at 590 nm. Results are presented as mean ± range from two independent experiments (left), or as mean ± SD from three independent experiments (right). **P <0.01. (B) Control MSCs show a polarized cell shape at the single‐cell level in a Wnt5a‐dependent manner. Cells were transfected with the indicated shRNA and a GFP expression vector and then plated on Matrigel for 2 h with control or Wnt5a‐conditioned medium, fixed, and stained for F‐actin and nucleus (with Dapi). (C) At least 100 GFP‐positive cells were counted for each condition, and cells with polarized actin were represented as percentage of total cells. Results are presented as mean ± SD from three independent experiments. **P <0.01. (D) IEC‐18 cells were transfected with the indicated shRNA and a GFP expression vector and stained for F‐actin. The percentage of GFP‐positive cells showing cortical actin was represented as above. Results are presented as mean ± range from two independent experiments.

Wnt5a also promotes other cellular responses, such as increased cell invasion (Kurayoshi *et al*., 2006). Elimination of p120‐catenin, CK1ε, or Ror2 prevented the upregulation in HEK293T invasion caused by Wnt5a (Fig. 6A, left); this result was also reproduced in MSCs (Fig. 6A, right). Another cellular response to Wnt5a is the polarization of cortical actin in single cells (Gon *et al*., 2013). Transfection of shRNA specific for Ror2, CK1ε, or p120‐catenin led to a very substantial downregulation of the percentage of cells exhibiting a polarized distribution of cortical actin upon Wnt5a addition (Fig. 6B, C). Similar results were also obtained in epithelial IEC cells, which also asymmetrically distributed cortical actin upon Wnt5a stimulation (Fig. 6D and Fig. S13).

## 4 Discussion

In this work, we have analyzed the signaling pathway stimulated by Wnt5a that – in contrast to the canonical Wnt pathway – does not increase β‐catenin levels. We confirmed previous results indicating that Wnt5a requires CK1ε to stimulate Dvl2 phosphorylation (Bryja *et al*., 2007) and Dvl2 binding to Ror2 co‐receptor (Witte *et al*., 2010). We demonstrated that Wnt5a ligand activates CK1ε through the action of the regulatory subunit of phosphatase PP2A, PR61ε, which is constitutively bound to Fz. Moreover, we determined that the presence of Ror2 in the plasma membrane is controlled by its binding to two proteins, CK1ε and p120‐catenin; these interactions require different sequences in Ror2. Binding of p120‐catenin maintains Ror2 in the membrane, whereas binding of CK1ε controls the stability of Ror2, preventing its lysosomal degradation.

**Figure 7:**
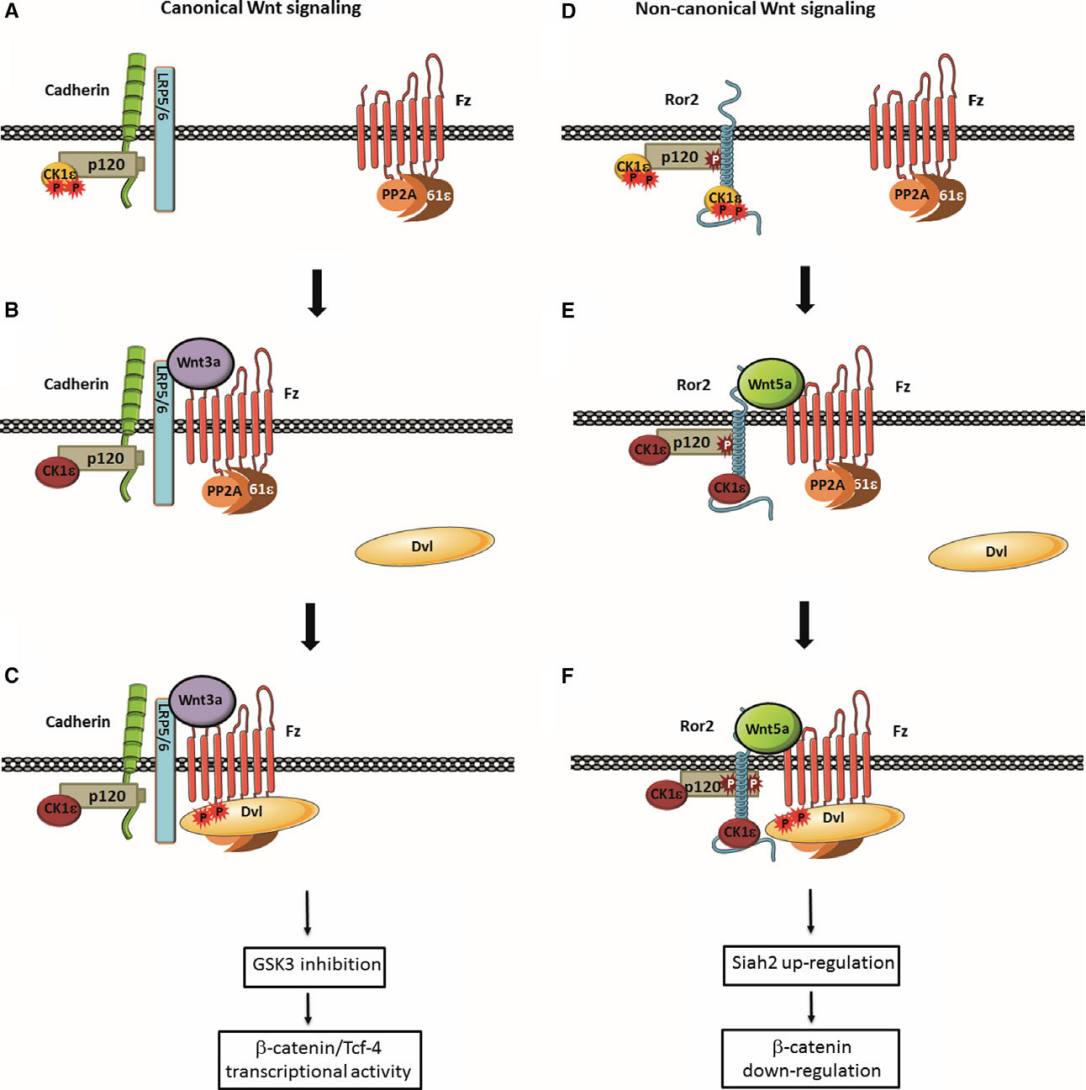
Model for the initiation of Ror2‐dependent noncanonical Wnt signaling. In the canonical Wnt pathway, Ser‐phosphorylated and inactive CK1ε (orange) binds to the LRP5/6 co‐receptor through cadherin and p120‐catenin (A); upon Wnt3a stimulation, PP2A, associated with Fz through the PR61ε regulatory subunit, becomes closer to LRP5/6‐bound CK1ε, allowing it to dephosphorylate and thus activate this kinase (B). Active CK1ε (dark red) phosphorylates either Dvl2, Fz, or both, thereby facilitating the interaction of Dvl2 with the receptor complex (C) and enabling the further reactions of this pathway, which lead to GSK3 inactivation and stimulation of β‐catenin/TCF‐4 transcriptional activity. Wnt5a activates noncanonical signaling using the same receptor but a different co‐receptor – namely, Ror2, which interacts with CK1ε both directly and indirectly through p120‐catenin (D). Association with p120‐catenin requires tyrosine phosphorylation of this protein and protects Ror2 from clathrin‐mediated internalization. Similar to the canonical Wnt pathway, Wnt5a‐induced assembly of the Fz–Ror2 complex enables CK1ε dephosphorylation and activation by PP2A (E), recruitment of Dvl2 to the complex (F) and an increase of p120‐catenin Tyr phosphorylation, which enhances its interaction with Ror2. Dvl2 binding to the complex is required for downstream signaling events, such as Rac1 and JNK activation, Siah2 expression, and β‐catenin downregulation.

Compared to canonical Wnt, much less is known about the noncanonical signaling pathway. However, it is clear that both pathways involve some common elements, such as Fz receptors, CK1ε and Dvl2 phosphoprotein (Grumolato *et al*., 2010). According to our model (Fig. 7), CK1ε is constitutively associated with the two specific co‐receptors for canonical and noncanonical Wnts. p120‐catenin is required for the association of CK1ε with the canonical Wnt co‐receptor LRP5/6; p120‐catenin‐bound CK1ε interacts with LRP5/6 through the association of both proteins with cadherin (Casagolda *et al*., 2010) (Fig. 7A). In contrast, CK1ε directly interacts with the noncanonical co‐receptor Ror2 (see Fig. 3A and Kani *et al*., 2004; Fig. 7D). Therefore, upon binding of the Wnt5a ligand, the Fz‐bound PR61ε regulatory subunit gets closer to Ror2‐associated CK1ε, thus facilitating the dephosphorylation and activation of this kinase by PP2A (Fig. 7E).

Subsequently, CK1ε promotes Dvl2 binding to Fz and oligomerization, although the mechanism is still under discussion (Fig. 7C, F). CK1ε might phosphorylate Fz, enhancing the affinity of this protein by Dvl2 (Tauriello *et al*., 2012). It might also participate in Dvl2 phosphorylation, although it is still not clear if this modification is required for Dvl2 polymerization (Bernatík *et al*., 2011, 2014; González‐Sancho *et al*., 2013). Another alternative that is not mutually exclusive is that the main substrate of CK1ε is Ror2 itself, and that phosphorylation of this protein increases its activity on other substrates required for downstream signals (Kani *et al*., 2004).

CK1ε not only transmits the Ror2 action but, through its direct interaction, protects Ror2 from lysosomal‐dependent degradation (Fig. 4). The CK1ε binding site was previously mapped to the Ror2 C‐terminal Pro‐rich domain by Minami and coworkers (Kani *et al*., 2004); this domain contains a lysine (K858) predicted to be ubiquitinated. Some receptor tyrosine kinases, such as EGF receptor, are regulated by monoubiquitination and subsequent lysosomal degradation (Levkowitz *et al*., 1998). It is possible that binding of CK1ε is interfering with the action of the E3 ligase responsible for this modification, providing an explanation for Ror2 stabilization.

Besides being controlled by CK1ε, the presence of Ror2 on the membrane is also controlled by p120‐catenin, which also directly binds to Ror2. p120‐catenin associates with Ror2 through its aa 102–350, a sequence present in p120‐catenin isoforms 1–3 but not in isoform 4 (Kourtidis *et al*., 2013). The p120‐catenin/Ror2 association is constitutive, as it is detected in nonstimulated cells, although it is enhanced by Wnt5a, depending on tyrosine phosphorylation of p120‐catenin by Ror2 or by an associated protein kinase. This increased p120‐catenin/Ror2 interaction after Wnt5a stimulation also upregulates binding of CK1ε to Ror2 (see Figs 3A and 4A). It remains to be established whether this increased CK1ε binding mediated by p120‐catenin contributes to Wnt5a‐activation of this protein kinase.

Through its binding, p120‐catenin also stabilizes cadherin at the plasma membrane and prevents its internalization (Davis *et al*., 2003; Kourtidis *et al*., 2013). Similar to the interaction site on cadherin, p120‐catenin binds to the Ror2 juxtamembrane sequence, a different domain to that binding to CK1ε. It remains to be determined whether, as for cadherins (Nanes *et al*., 2012; Xiao *et al*., 2005), binding to p120‐catenin blocks the access of AP2 to an endocytic signal in Ror2. In any case, our results indicate that p120‐catenin regulates the endocytosis of proteins other than cadherins.

It is important to remark that the noncanonical Wnt model presented in Fig. 7 is limited to the pathway dependent on Ror2. Several signaling cascades have been reported to be stimulated by Wnt5a and other Wnt ligands acting through pathways that do not stimulate β‐catenin‐dependent transcription (Kikuchi *et al*., 2012). In many cases, it remains to be established how many of these pathways require Ror2. At least in our cells, this molecule is dispensable for ERK2 activation (see Fig. 1D); accordingly, p120‐catenin and CK1ε are not needed either for the stimulation of this kinase (Figs S4A and S5A). Therefore, our model predicts that as Ror2, CK1ε, and p120‐catenin are so closely interconnected, the Wnt5a‐induced reactions requiring one of these elements are also affected by the elimination of the other two.

In conclusion, the results presented in this study indicate that p120‐catenin and CKIε interact with Ror2 and are essential for Ror2‐dependent, noncanonical Wnt signaling. CK1ε is activated by this pathway and is required for Dvl2 recruitment to Fz. Moreover, association between both p120‐catenin and CK1ε is necessary for maintaining Ror2 at the plasma membrane.

## Author contributions

MD and AGH designed and directed the research. JC, BdV‐P, AV, GF, and MV performed the research. RP contributed to essential reagents. AGH and MD wrote the article, with contributions from the rest of the authors.

## Supporting information


**Fig. S1.** A Wnt5a antibody prevents the stimulation of the JNK2 and ERK2 serine kinases and the association of Fz with Dvl2 induced by Wnt5a.

**Fig. S2.** The tyrosine kinase inhibitor herbimycin affects Wnt5a signaling.

**Fig. S3.** CK1ε CRISPR cells contain unaltered levels of CK1δ.

**Fig. S4.** p120‐catenin deficiency prevents Wnt5a‐induced JNK2 phosphorylation and Rac1 activation.

**Fig. S5.** CK1ε deficiency prevents Wnt5a‐induced JNK2 phosphorylation and Rac1 activation.

**Fig. S6.** N‐cadherin is not required for Wnt5a signaling.

**Fig. S7.** Herbimycin decreases Wnt5a‐induced p120‐catenin phosphorylation and its interaction with Ror2.

**Fig. S8.** Ror2 interacts with p120‐catenin.

**Fig. S9.** Herbimycin promotes Ror2 internalization.

**Fig. S10.** CK1ε binds to the C‐terminal domain of Ror2.

**Fig. S11.** CK1ε depletion decreases Ror2 protein stability but not Ror2 RNA levels.

**Fig. S12.** Siah2 shRNA prevents β‐catenin downregulation caused by Wnt5a.

**Fig. S13.** Ror2, p120‐catenin and CK1ε are required for Wnt5a‐induced asymmetrical distribution of cortical actin in IEC‐18 cells.

**Table S1.** List of antibodies used in this work.
