# Supplementary figures and images for: CK1ε and p120‐catenin control Ror2 function in noncanonical Wnt signaling
Source: Mol Oncol. 2018 Mar 14;12(5):611–29. doi: 10.1002/1878-0261.12184 (PMC5928365; doi:10.1002/1878-0261.12184)

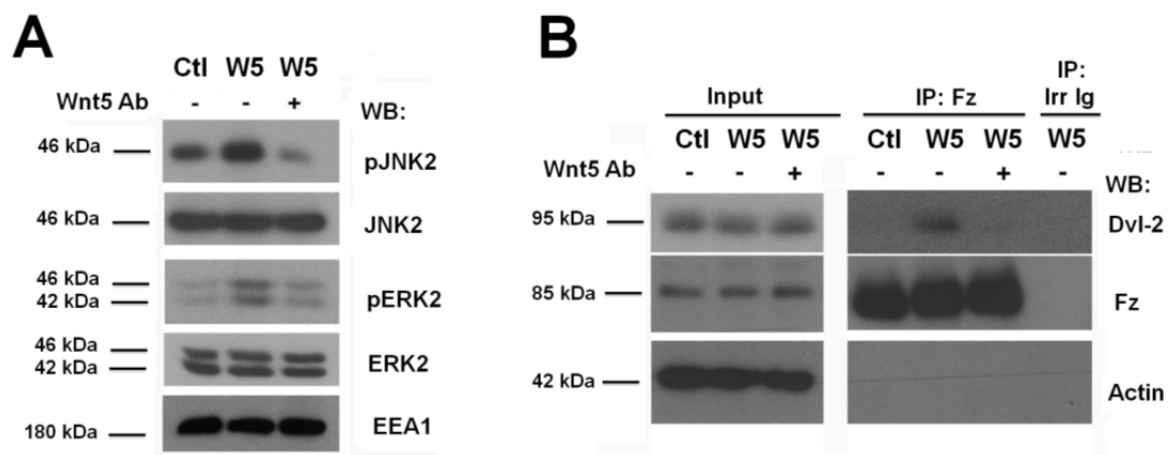

**Figure S1**

Supplement: Supplementary file 1 — Fig. S1. A Wnt5a antibody prevents the stimulation of the JNK2 and ERK2 serine kinases and the association of Fz with Dvl2 induced by Wnt5a. [file MOL2-12-611-s001.pdf]

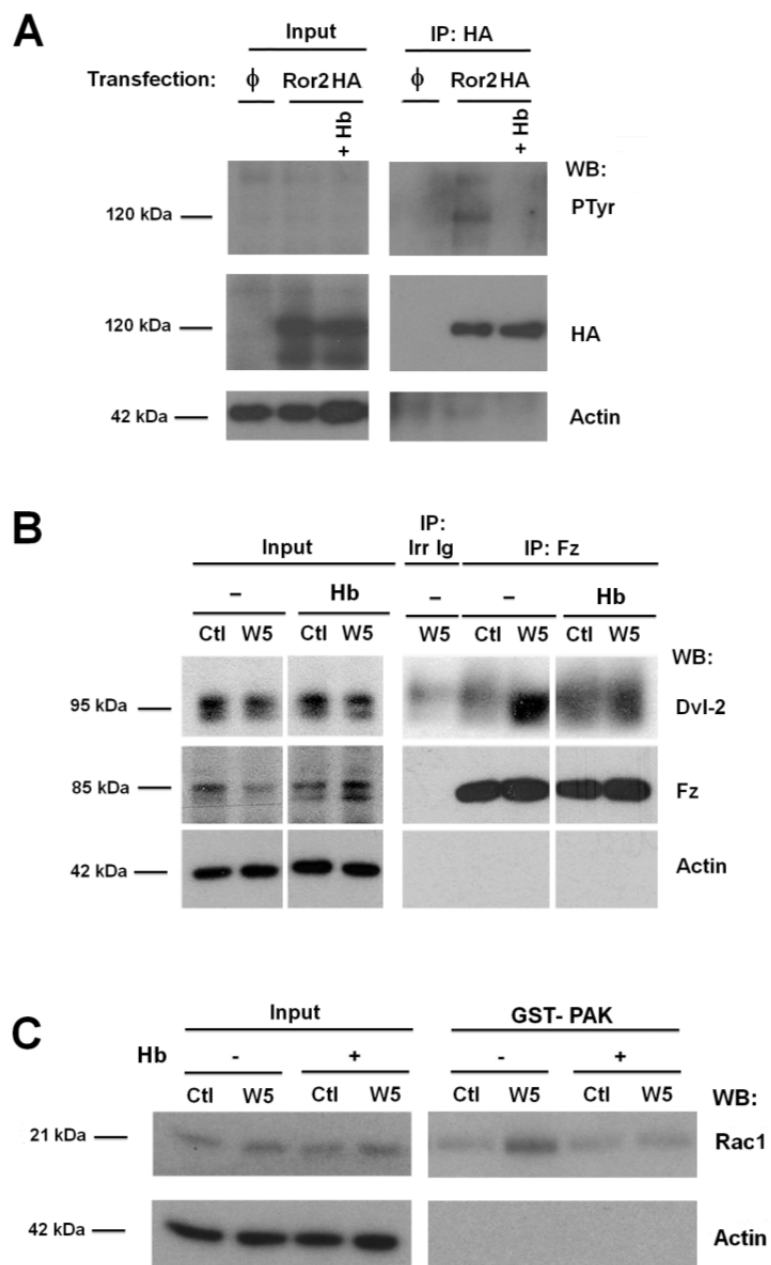

**Figure S2**

Supplement: Supplementary file 2 — Fig. S2. The tyrosine kinase inhibitor herbimycin affects Wnt5a signaling. [file MOL2-12-611-s002.pdf]

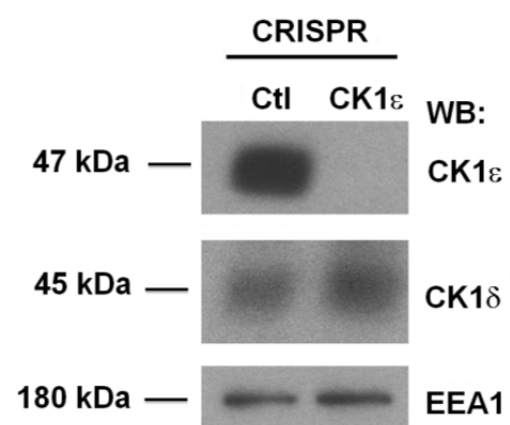

**Figure S3**

Supplement: Supplementary file 3 — Fig. S3. CK1ε CRISPR cells contain unaltered levels of CK1δ. [file MOL2-12-611-s003.pdf]

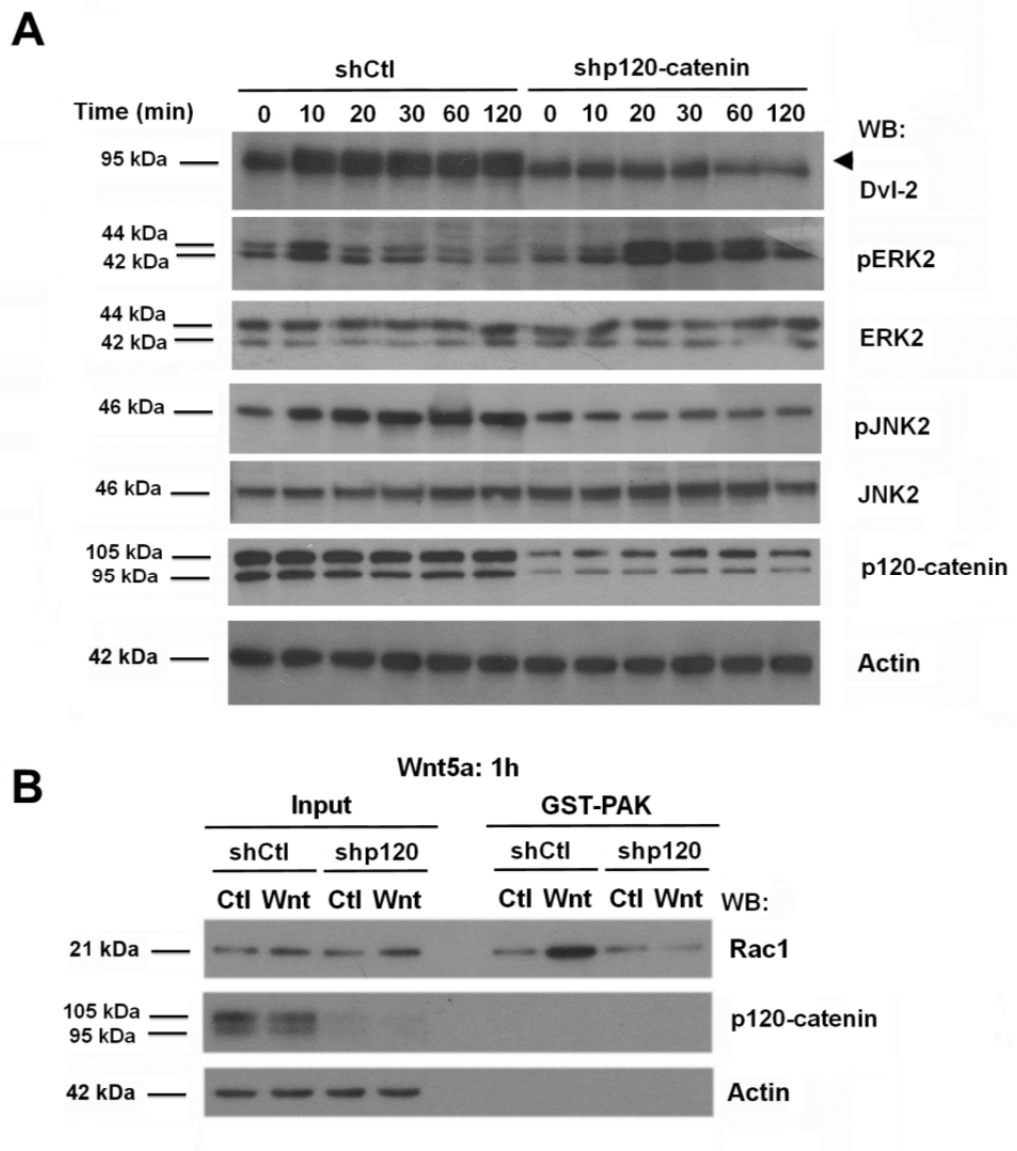

**Figure S4**

Supplement: Supplementary file 4 — Fig. S4. p120‐catenin deficiency prevents Wnt5a‐induced JNK2 phosphorylation and Rac1 activation. [file MOL2-12-611-s004.pdf]

**A**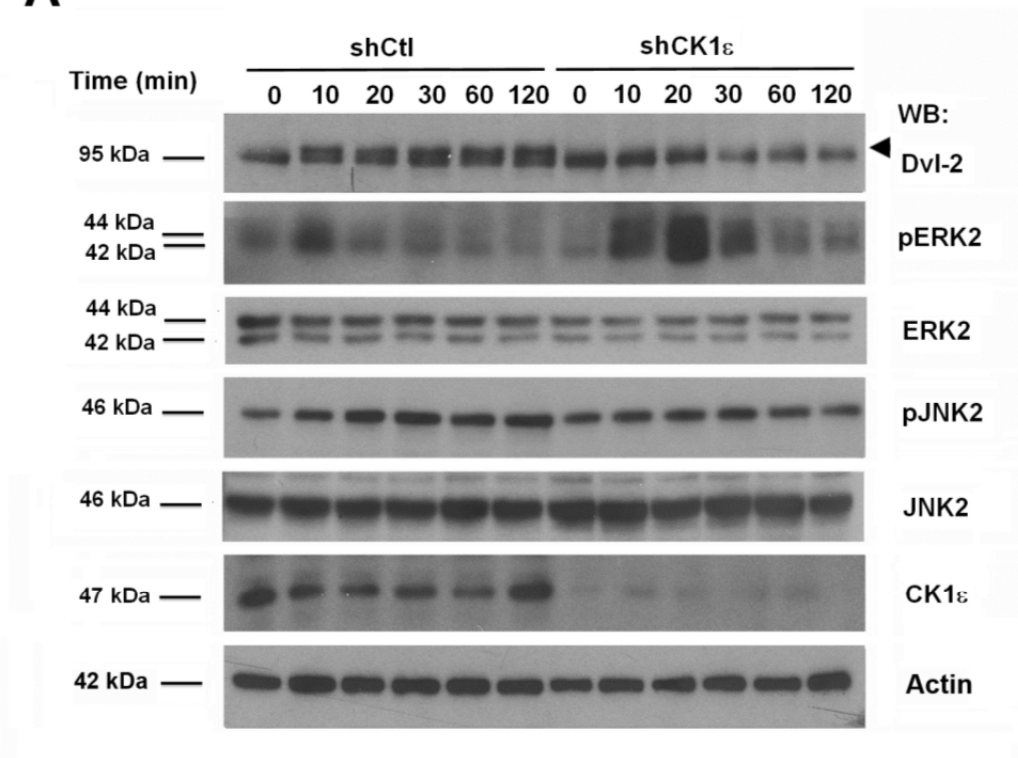**B**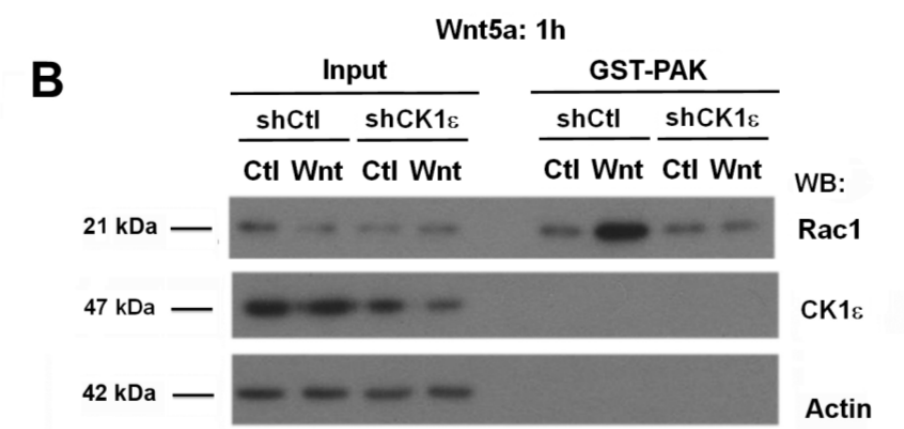**Figure S5**

Supplement: Supplementary file 5 — Fig. S5. CK1ε deficiency prevents Wnt5a‐induced JNK2 phosphorylation and Rac1 activation. [file MOL2-12-611-s005.pdf]

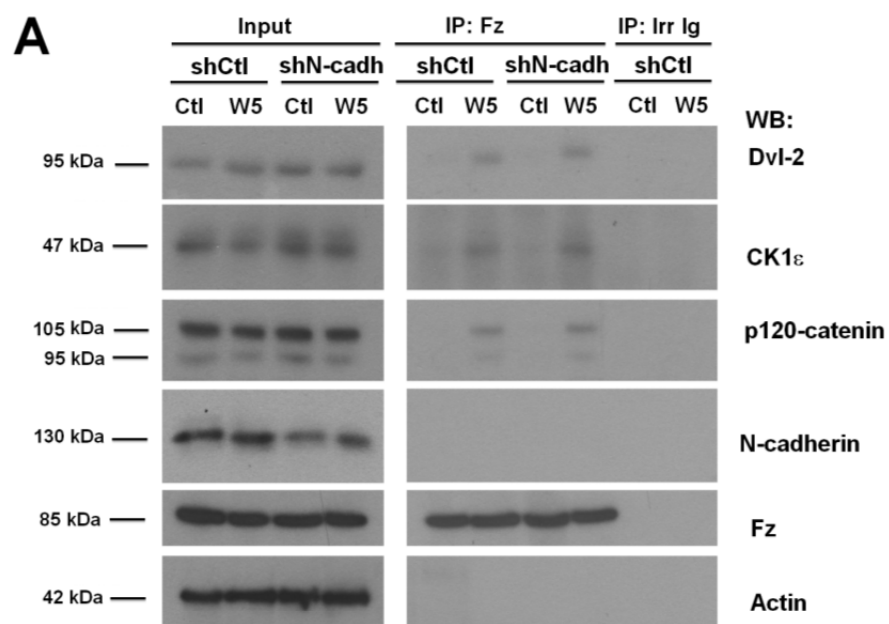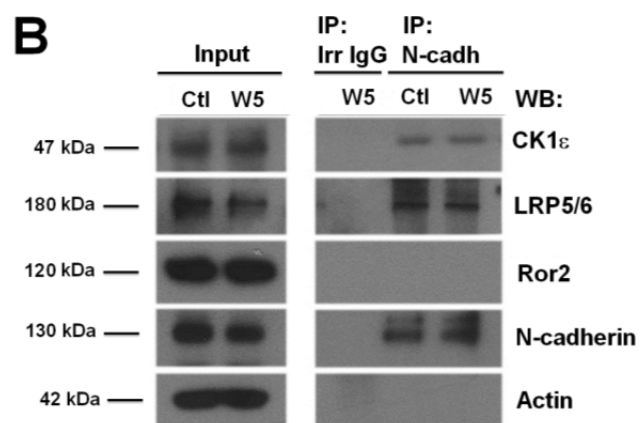

**Figure S6**

Supplement: Supplementary file 6 — Fig. S6. N‐cadherin is not required for Wnt5a signaling. [file MOL2-12-611-s006.pdf]

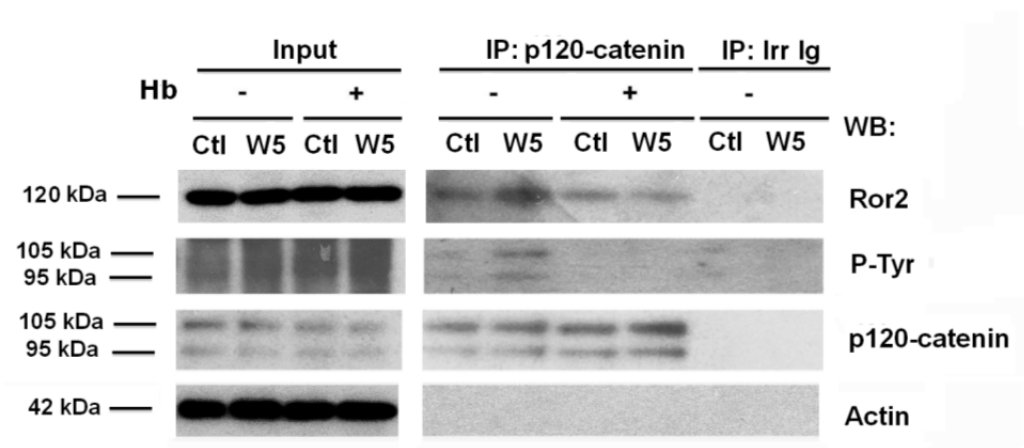

**Figure S7**

Supplement: Supplementary file 7 — Fig. S7. Herbimycin decreases Wnt5a‐induced p120‐catenin phosphorylation and its interaction with Ror2. [file MOL2-12-611-s007.pdf]

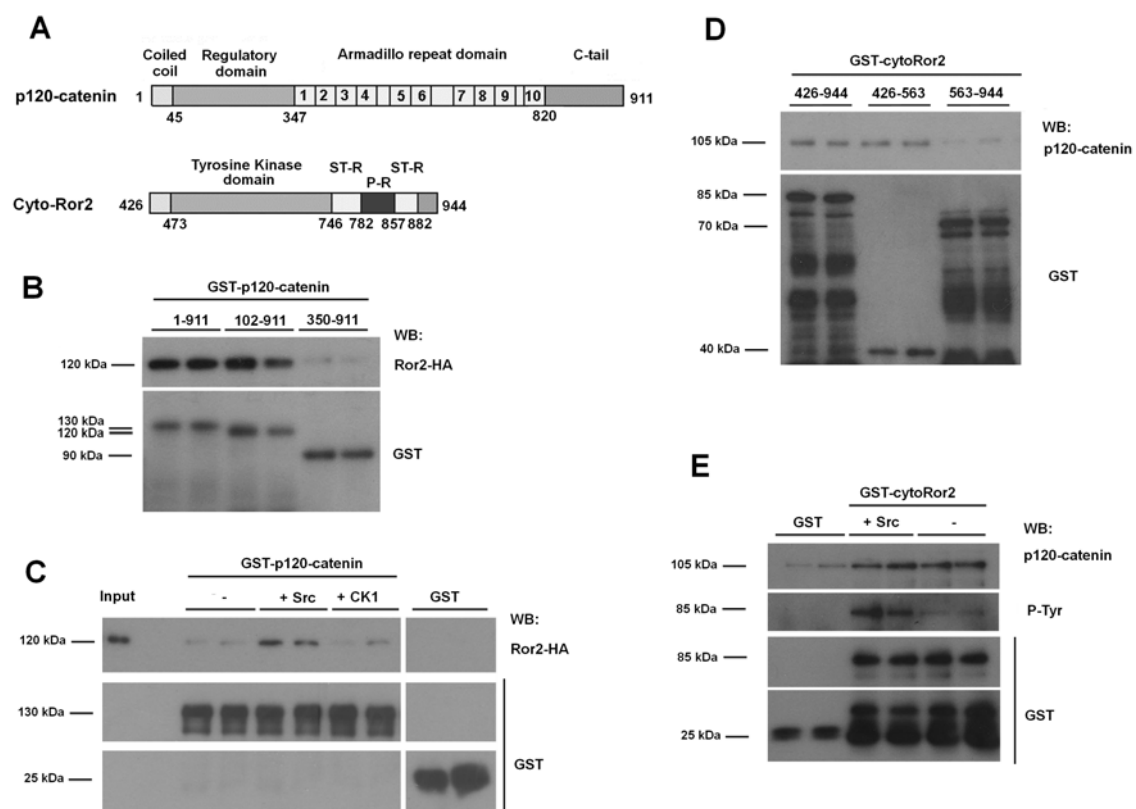

**Figure S8**

Supplement: Supplementary file 8 — Fig. S8. Ror2 interacts with p120‐catenin. [file MOL2-12-611-s008.pdf]

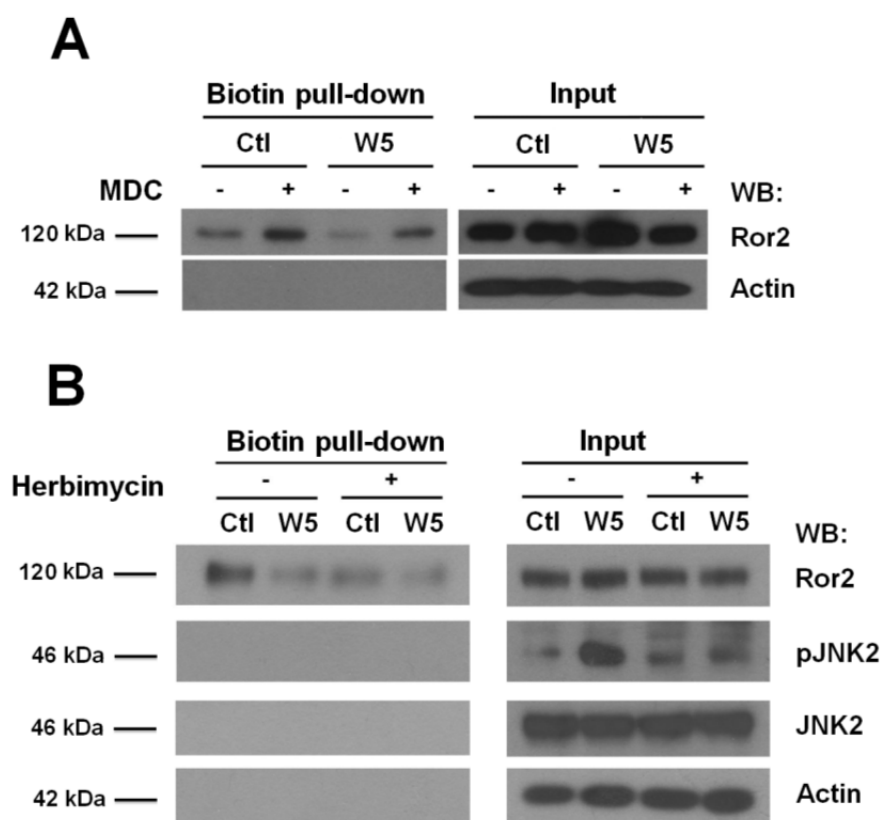

**Figure S9**

Supplement: Supplementary file 9 — Fig. S9. Herbimycin promotes Ror2 internalization. [file MOL2-12-611-s009.pdf]

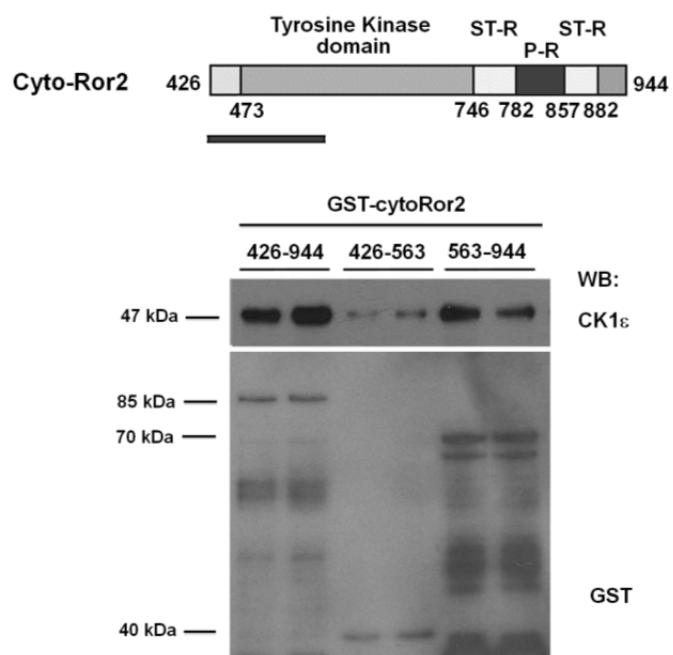

**Figure S10**

Supplement: Supplementary file 10 — Fig. S10. CK1ε binds to the C‐terminal domain of Ror2. [file MOL2-12-611-s010.pdf]

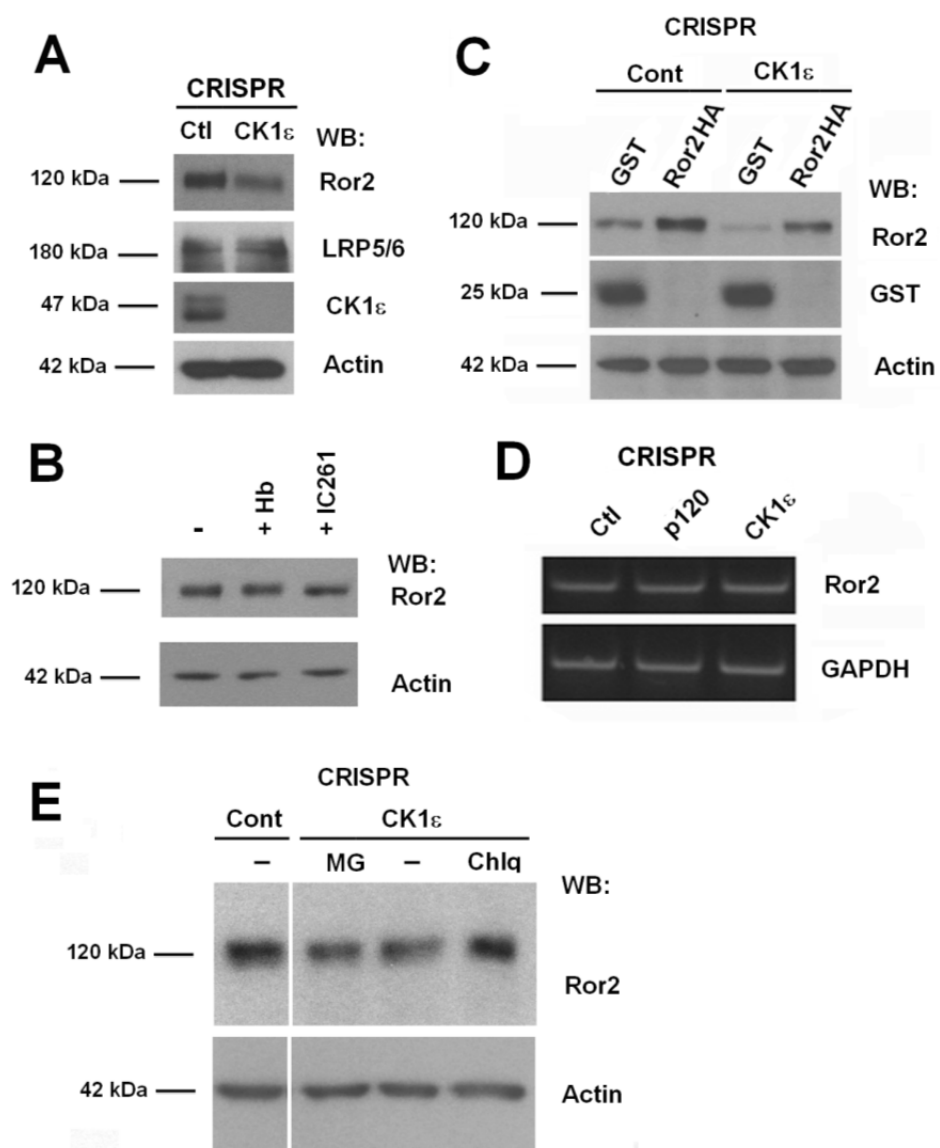

**Figure S11**

Supplement: Supplementary file 11 — Fig. S11. CK1ε depletion decreases Ror2 protein stability but not Ror2 RNA levels. [file MOL2-12-611-s011.pdf]

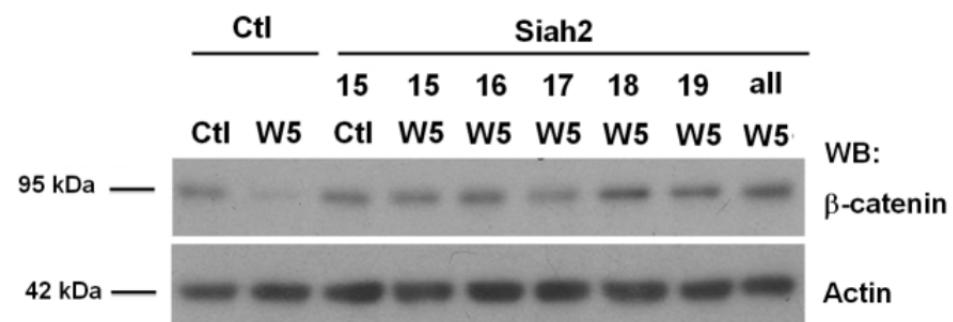

**Figure S12**

Supplement: Supplementary file 12 — Fig. S12. Siah2 shRNA prevents β‐catenin downregulation caused by Wnt5a. [file MOL2-12-611-s012.pdf]

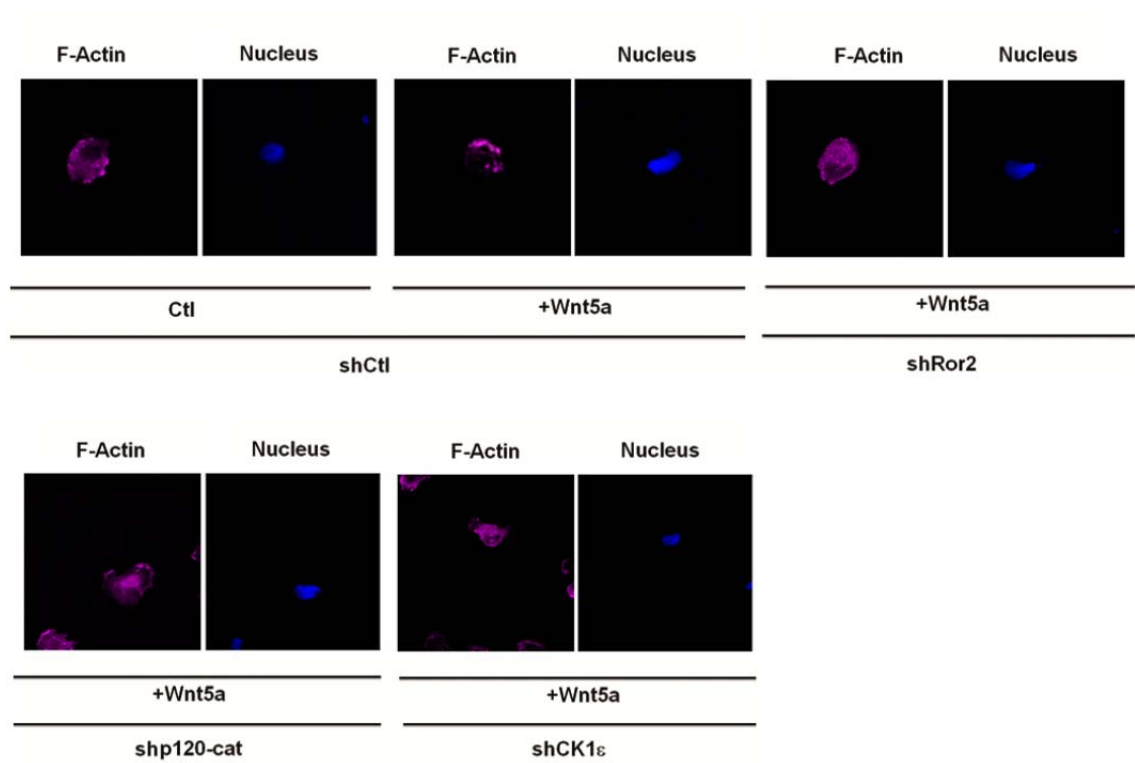

**Figure S13**

Supplement: Supplementary file 13 — Fig. S13. Ror2, p120‐catenin and CK1ε are required for Wnt5a‐induced asymmetrical distribution of cortical actin in IEC‐18 cells. [file MOL2-12-611-s013.pdf]
