# Supplementary material for: CK1ε and p120‐catenin control Ror2 function in noncanonical Wnt signaling
Source: Mol Oncol. 2018 Mar 14;12(5):611–29. doi: 10.1002/1878-0261.12184 (PMC5928365; doi:10.1002/1878-0261.12184)
Supplement: Supplementary file 14 — Table S1. List of antibodies used in this work. [file MOL2-12-611-s016.docx]

| **Antibody** | **Supplier** | **Reference** | **Assay** |
| --- | --- | --- | --- |
| Axin | Santa Cruz Biotechnologies | sc-14029 | WB |
| β-Actin | Sigma | A5441 | WB |
| β-catenin | BD Biosciences | 610153 | WB |
| CK1γ | Abcam | ab64829 | WB/IP |
| CK1ε | BD Biosciences | 610445 | WB/IP |
| CK1δ | Abcam | ab48031 | WB |
| Dvl2 | Cell Signaling | 3216 | WB |
| EEA1 | BD Biosciences | 610457 | WB |
| ERK1/2 | Cell Signaling | 9107 | WB |
| Fz (total) | Santa Cruz Biotechnologies | sc-9169 | WB/IP |
| Fz2 | Abcam | ab52565 | WB/IP |
| GST | GE Healthcare | 27457701 | WB |
| HA | Roche | 11867423001 | WB/IP |
| JNK2 | Abcam | ab178953 | WB |
| LRP6 | Santa Cruz Biotechnologies | sc-15399 | WB |
| Na^+^, K^+^ ATPase | Abcam | ab7671 | WB |
| N-cadherin | BD Biosciences | 610921 | WB/IP |
| p120-catenin | BD Biosciences | 610134 | WB/IP |
| Phospho ERK 1/2 (Thr202/Tyr204) | Cell Signaling | 4370 | WB |
| Phospho JNK (Thr183/Tyr185, Thr221/Tyr223) | Millipore | 07-175 | WB |
| Phospho p120-catenin (Ser 268) | Santa Cruz Biotechnologies | sc-293000 | WB |
| Phospho LRP5/6 (Thr1490) | Cell Signaling | 2568 | WB |
| Phospho Tyrosine | BD Biosciences | 610000 | WB/IP |
| PR61ε | Jin *et al*. 2009 |  | WB |
| Rac1 | BD Biosciences | 610650 | WB |
| Ror2 | Santa Cruz Biotechnologies | sc-374174 | WB/IP |
| Wnt5a | R&D System | AF645 | Wnt5a-specific neutralization |

**Table S1: List of antibodies used in this work**. The table indicates the source, catalogue number and use in Western Blot (WB) or immunoprecipitation (IP) experiments of all the antibodies employed in this article.

Jin Z, Shi J, Saraf A, Mei W, Zhu GZ, Strack S and Yang J (2009) The 48-kDa alternative translation isoform of PP2A:B56epsilon is required for Wnt signaling during midbrain-hindbrain boundary formation. *J Biol Chem* **284**, 7190–7200.
