# Supplementary material for: CK1ε and p120‐catenin control Ror2 function in noncanonical Wnt signaling
Source: Mol Oncol. 2018 Mar 14;12(5):611–29. doi: 10.1002/1878-0261.12184 (PMC5928365; doi:10.1002/1878-0261.12184)
Supplement: Supplementary file 15 [file MOL2-12-611-s014.pdf]

Supplementary Information for

CK1ε and p120-catenin control Ror2 function in non-canonical Wnt signaling

Josué Curto, Beatriz Del Valle-Pérez, Aida Villarroel, Guillem Fuertes, Meritxell Vinyoles, Raúl Peña, Antonio García de Herreros and Mireia Duñach

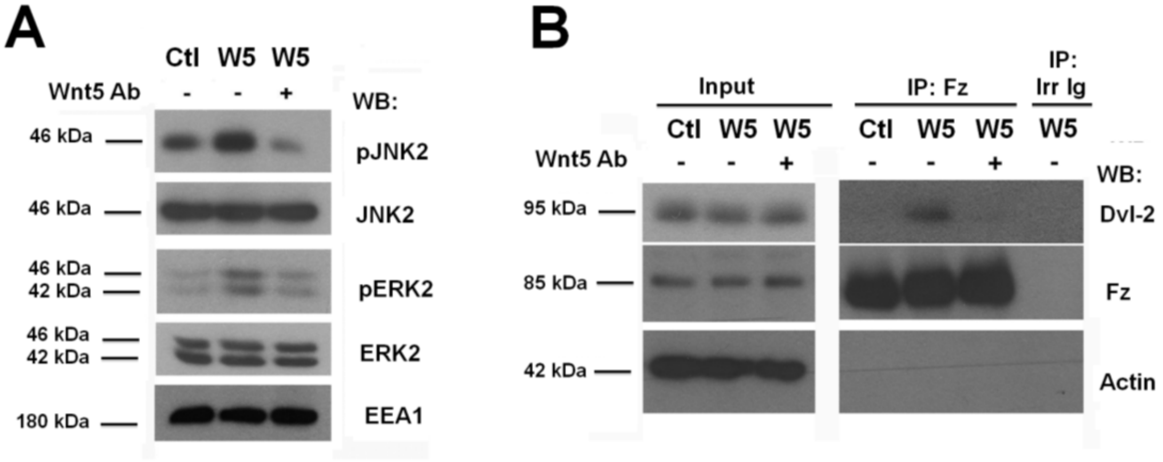

Fig. S1. A Wnt5a antibody prevents the stimulation of JNK2 and ERK2 Serine kinases and the association of Fz with Dvl-2 induced by Wnt5a. Control or Wnt5a-condtioned medium were incubated with a Wnt5a mab or an irrelevant IgG (both 2 $\mu g.mL^{-1}$) for 1 h. HEK293T cells were treated for 10 min with the indicated conditional medium. Extracts were analyzed by WB with the indicated antibodies (**A**). JNK2 and ERK2 phosphorylation were determined with anti-phospho antibodies against JNK and anti ERK (see Fig. 1). (**B**) Fz2 was immunoprecipitated and associated Dvl2 was detected by WB.

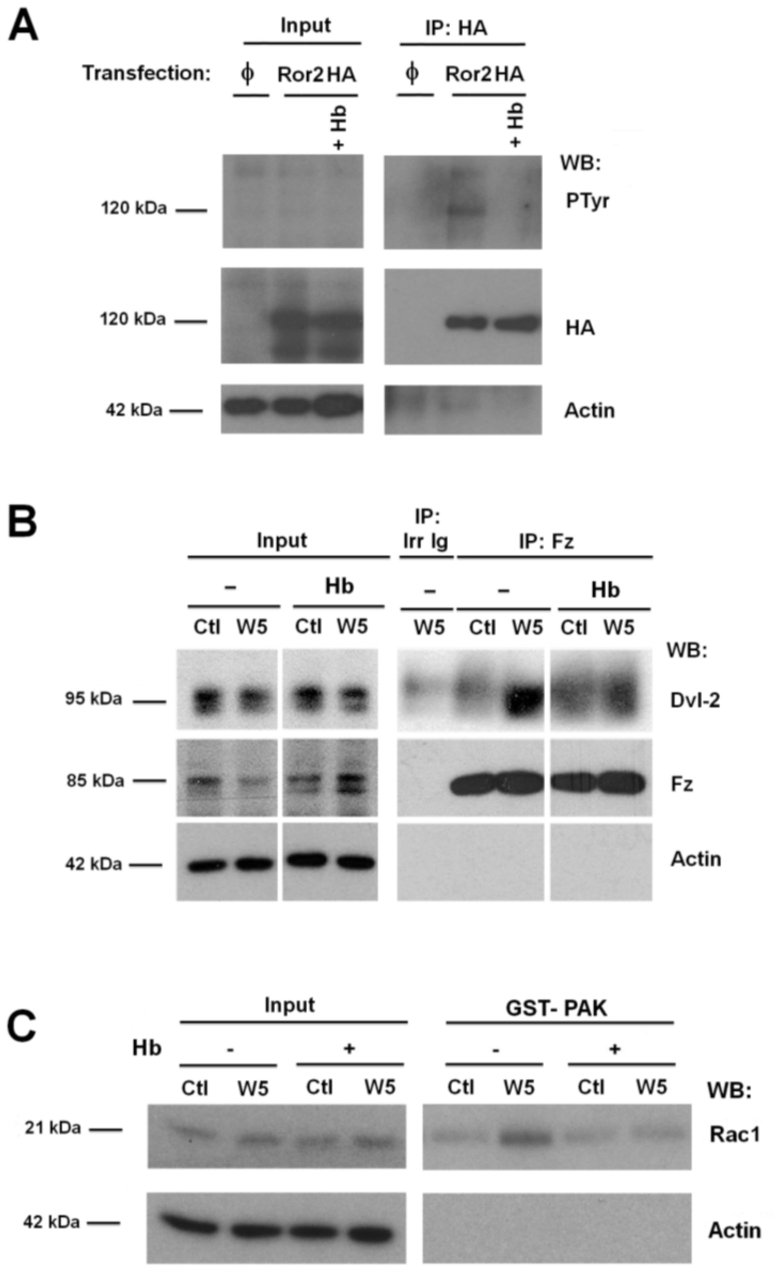

Fig. S2. The tyrosine kinase inhibitor Herbimycin affects Wnt5a signaling. When indicated, HEK293T cells were pretreated for 1 hour with Herbimycin (Hb, 20 ng.mL^{-1}) and stimulated with control or Wnt5a-conditioned medium for additional 5 min. (**A**) Ror2-HA was immunoprecipitated from HEK293T cell extracts overexpressing Ror2-HA and immunocomplexes were analyzed by WB. (**B, C**) HEK293T cells were pretreated with inhibitors; then, cells were stimulated with control or Wnt5a-conditioned medium for one additional hour supplemented with the inhibitor when indicated. Fz2 was immunoprecipitated from cell extracts and associated Dvl2 detected by WB (B), or GST–PAK pull-down assays were performed and active Rac1 analyzed by WB (C).

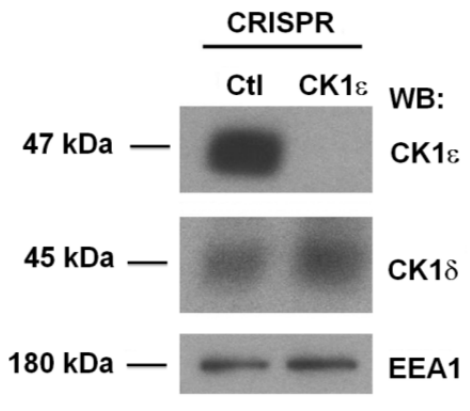

Fig. S3. CK1ε CRISPR cells contain unaltered levels of CK1δ. Levels of the indicated proteins were determined by WB in total extracts from control or CK1ε CRISPR cells.

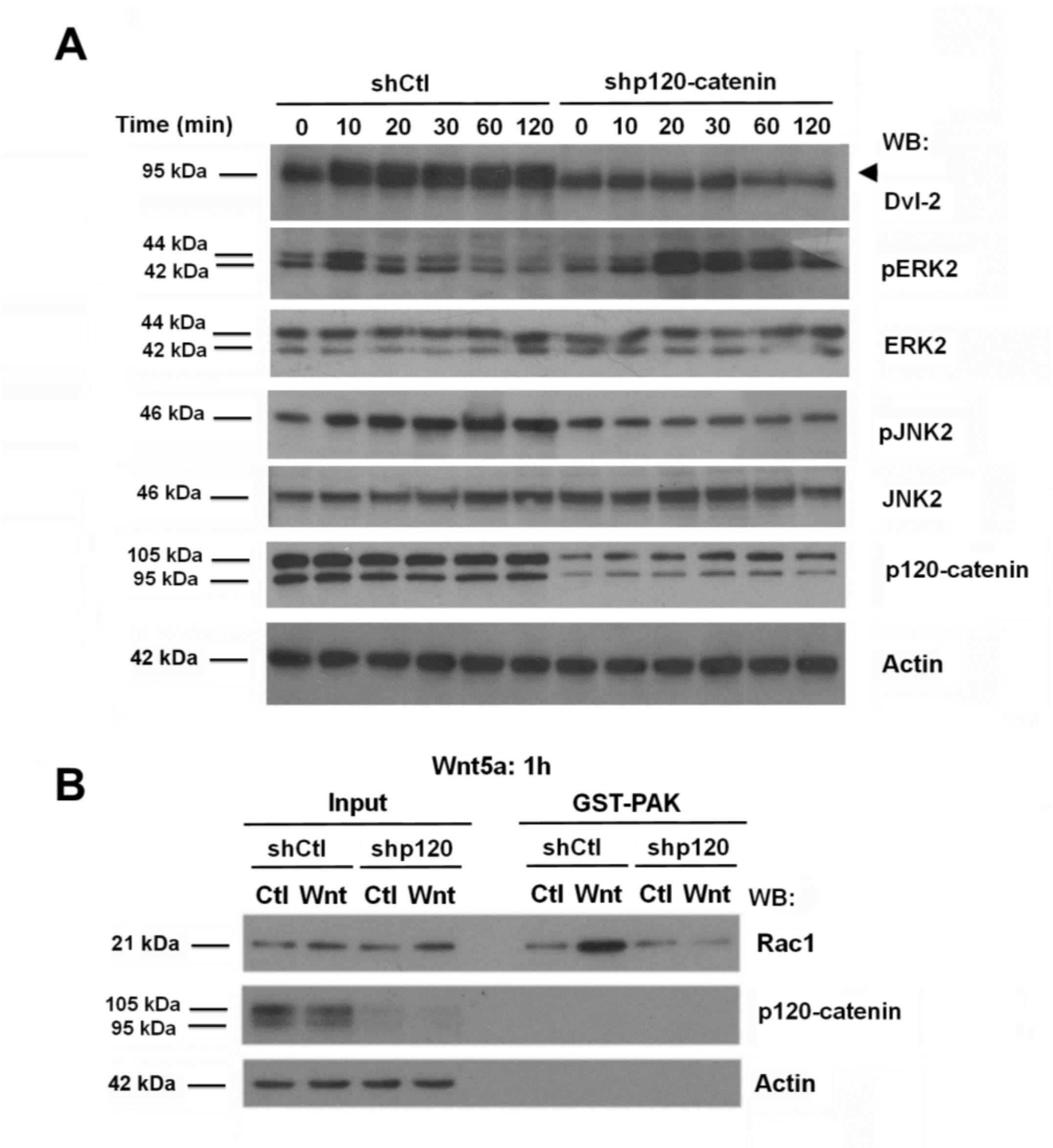

Fig. S4. p120-catenin deficiency prevents Wnt5a-induced JNK2 phosphorylation and Rac1 activation. HEK293T cells were depleted of p120-catenin using specific shRNA or a scrambled shRNA as a control. (**A**) Cells were treated with control or Wnt5a-conditioned medium for the indicated times and total levels of phosphorylated proteins were determined by WB with specific antibodies. The arrow indicates phosphorylated Dvl2. (**B**) Cells were treated for 1 h with control or Wn5a-conditioned medium. GST–PAK pull-down assays were performed and active Rac1 was detected by WB.

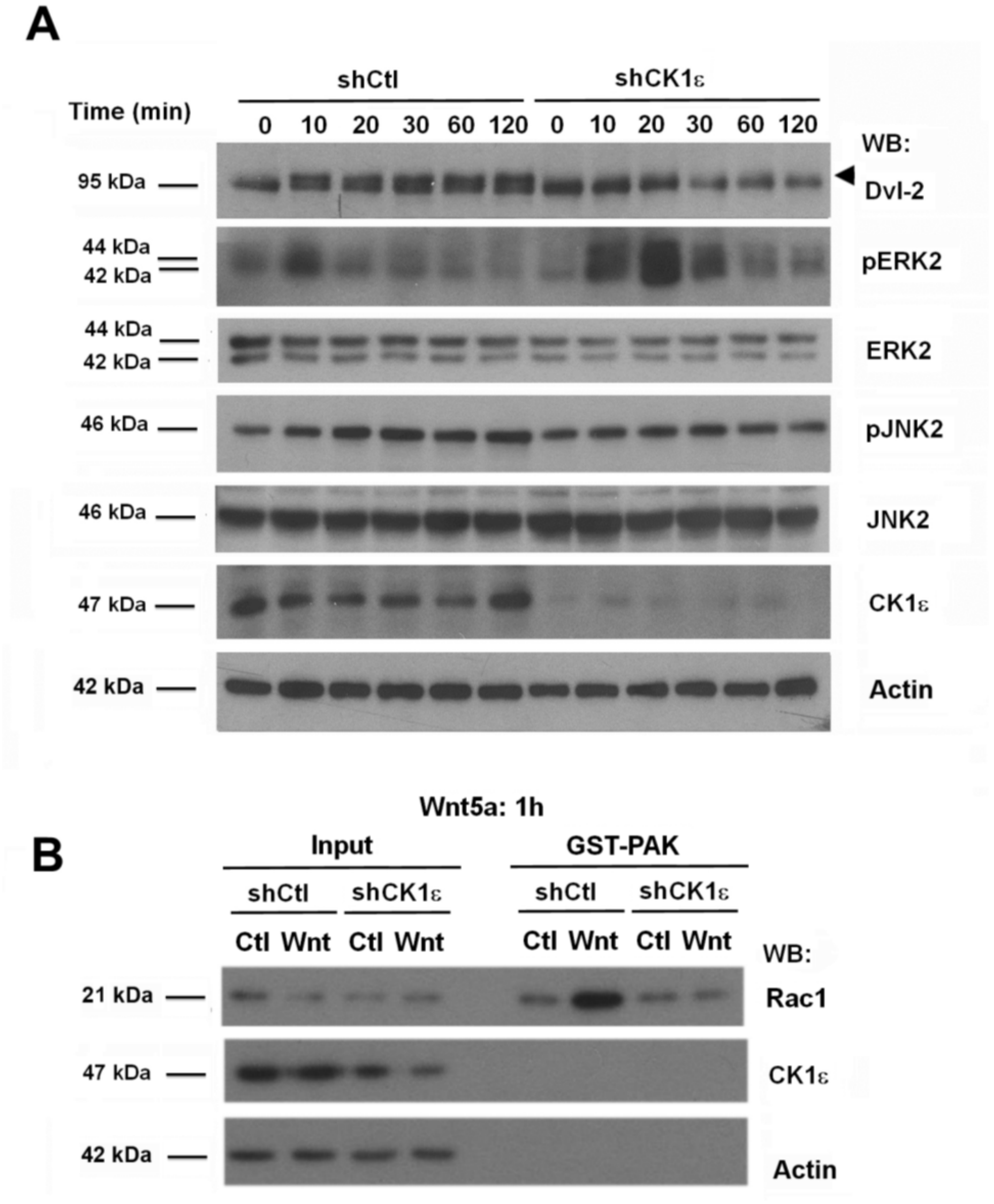

Fig. S5. CK1ε deficiency prevents Wnt5a-induced JNK2 phosphorylation and Rac1 activation. HEK293T cells were depleted of CK1ε using specific shRNA or a scrambled shRNA as a control. (**A**) Cells were treated with control or Wnt5a-conditioned medium for the indicated times and total levels of phosphorylated proteins were determined by WB with specific antibodies. The arrow indicates phosphorylated Dvl2. (**B**) Cells were treated for 1 h with control or Wn5a-conditioned medium. GST–PAK pull-down assays were performed and active Rac1 was detected by WB.

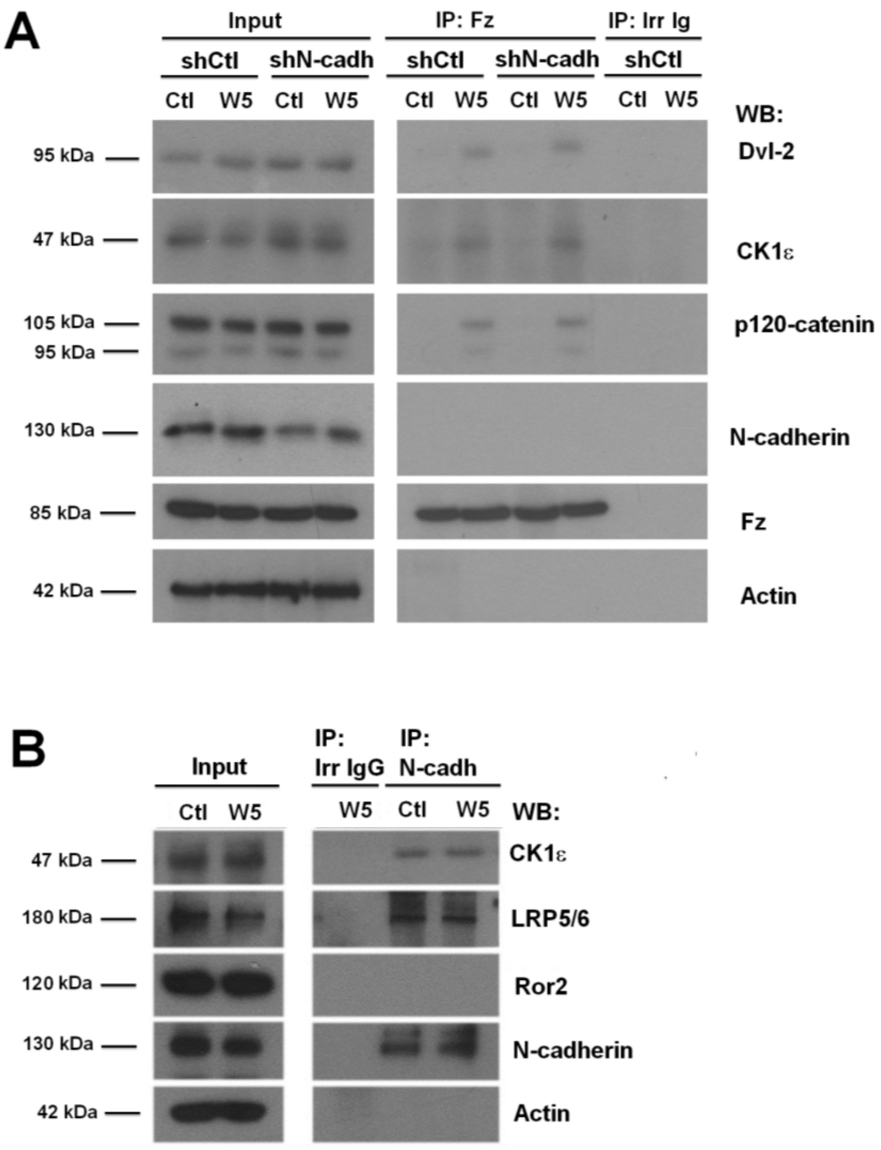

Fig. S6. N-cadherin is not required for Wnt5a signaling. (**A**) HEK293T cells were depleted of N-cadherin using a specific shRNA or a scrambled shRNA as a control. Cells were treated with control or Wnt5a-conditioned medium for 1h; Fz2 was immunoprecipitated from cell extracts and associated proteins were determined by WB. (**B**) N-cadherin was immunoprecipitated from extracts of cells treated with Wnt5a for 1 h when indicated. Immunocomplexes were analyzed by WB.

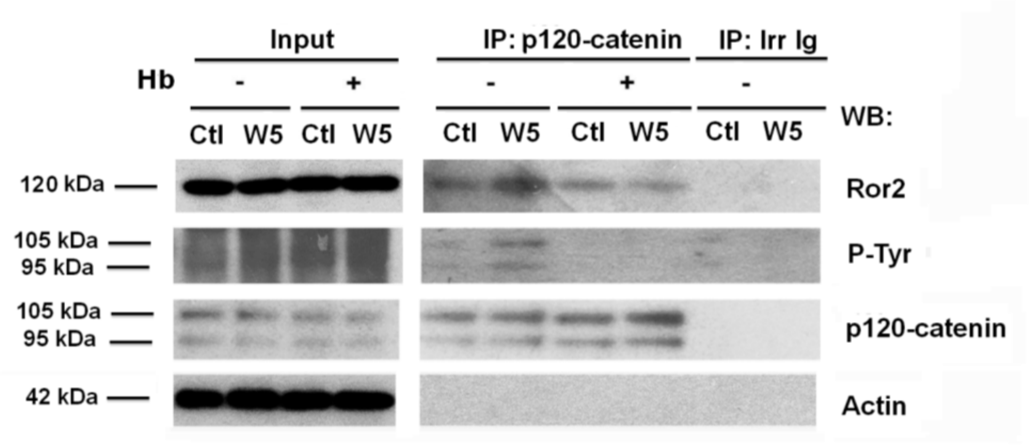

Fig. S7. Herbimycin decreases Wnt5a-induced p120-catenin phosphorylation and interaction with Ror2. When indicated HEK293T cells were pretreated for 1 h with Hb (20 ng.mL^{-1}) and then stimulated with control or Wnt5a-conditioned medium for additional 5 min. Cells were lysed and p120-catenin was immunoprecipitated. Tyrosine phosphorylated p120-catenin and associated Ror2 were analyzed by WB.

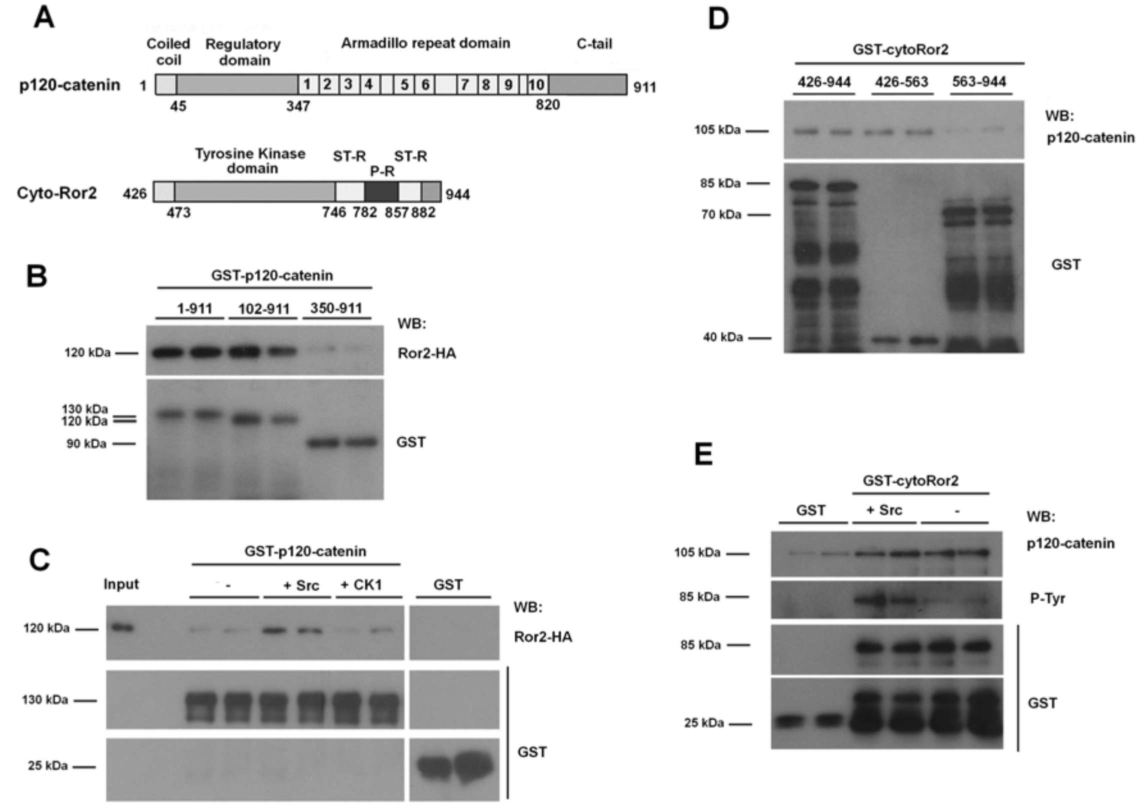

Fig. S8. Ror2 and p120-catenin interact. (**A**) Diagram of p120-catenin isoform 1 and the cytosolic region of Ror2 (cyto-Ror2) illustrating the different protein domains. ST-R and P-R indicate the Ser-Thr and Pro-rich domains. (**B**) GST–p120-catenin fusion proteins (7 pmol) were incubated with cells extracts overexpressing Ror2-HA and treated with Wnt5a-conditioned medium for 5 min. A pull-down assay was performed and bound Ror2-HA was analyzed by WB with anti-HA. (**C**) Full-length GST–p120-catenin (7 pmol) was in vitro phosphorylated with recombinant Src or CK1 kinases. A pull-down assay was performed by incubating the fusion proteins with cell extracts overexpressing Ror2-HA. Protein complexes were affinity purified and analyzed by WB. (**D**) Different GST–cytoRor2 fusion proteins (0.5 pmol) were incubated with recombinant p120-catenin (2 pmol). Protein complexes were affinity purified and analyzed by WB with anti-p120-catenin. (**E**) GST–cytoRor2 (amino acids 426-944) was in vitro phosphorylated with recombinant Src kinase for 2 h. A binding assay was performed by incubating 2 pmol of GST-cytoRor2 with 1 pmol of recombinant p120-catenin.

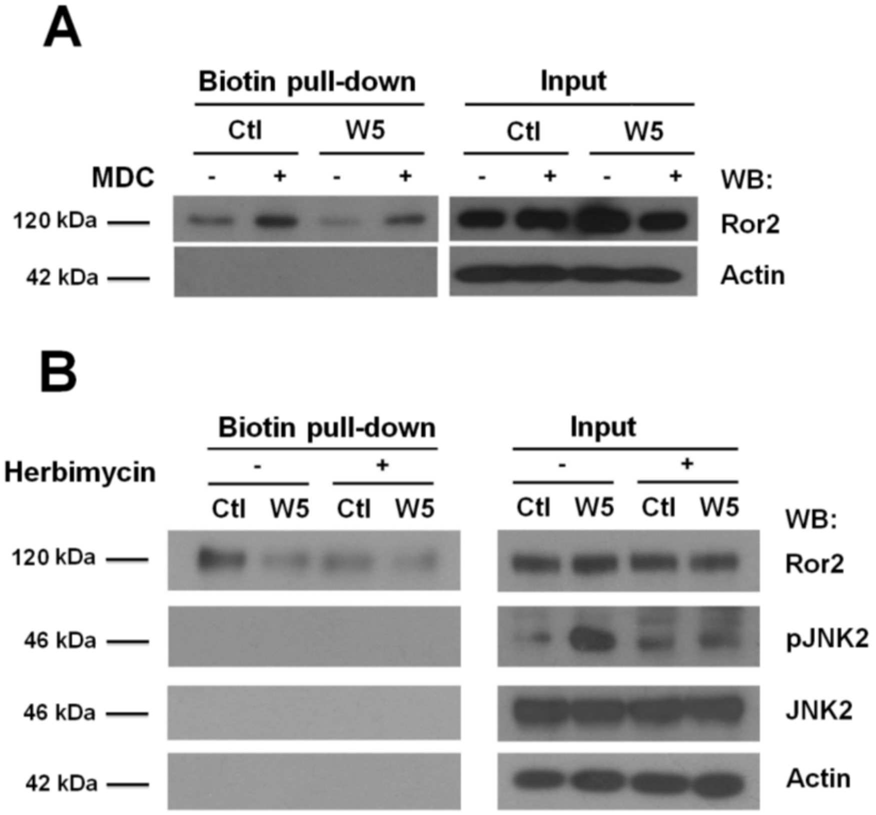

Fig. S9. Herbimycin promotes Ror2 internalization. HEK293T cells pretreated with monodansylcadaverine (MDC, 50 μM) for 30 min in (**A**) or Hb (20 ng.mL^{-1}) for 1 hour in (**B**). Then, cells were stimulated with control or Wnt5a-conditioned medium with the corresponding inhibitor for additional 20 min. Surface proteins were biotinylated and a pull-down assay was performed with NeutrAvidin Agarose. Biotinylated Ror2 and phosphorylated JNK were analyzed by WB with specific antibodies.

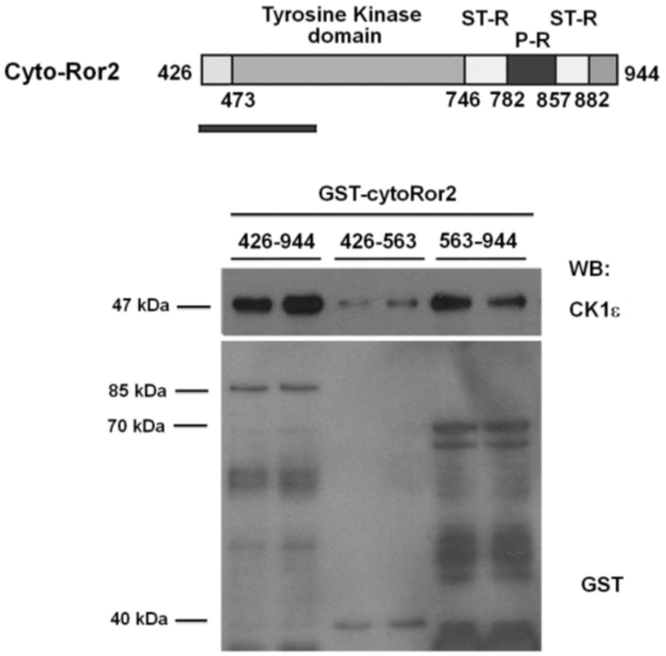

Fig. S10. CK1ε binds to the C-terminal domain of Ror2. Different GST–cytoRor2 fragments (0.5 pmol) were incubated with recombinant CK1ε (2 pmol). Protein complexes were affinity purified and analyzed by WB with anti-CK1ε. The bar under the diagram indicates the p120-catenin binding element in Ror2 as determined in Fig. S8.

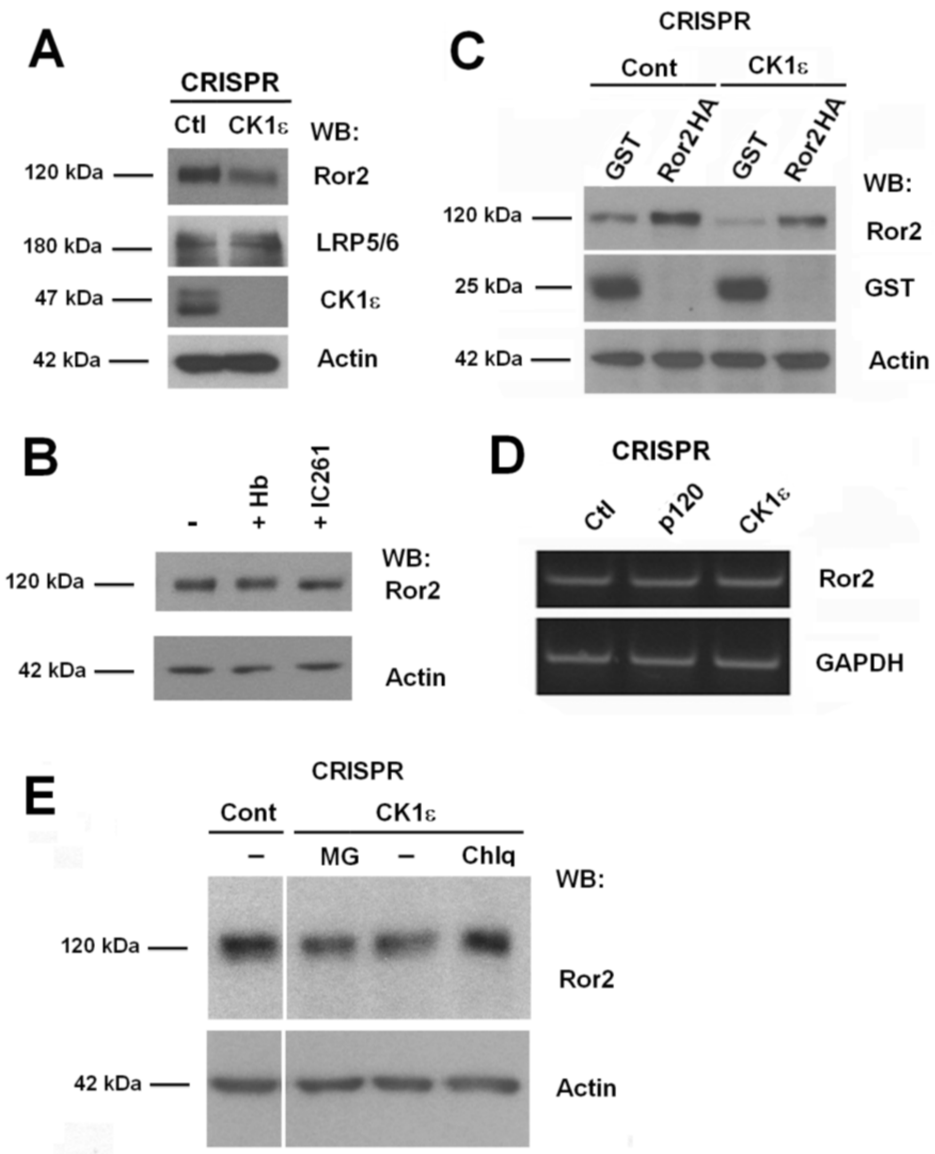

Fig. S11. CK1ε depletion decreases Ror2 protein stability but not Ror2 RNA levels. (**A**) Cell extracts from control or CRISPR CK1ε-depleted HEK293T cells were analyzed by WB. (**B**) HEK293T cells were treated with Hb (20 ng.mL^{-1}) or IC261 (15 μM) for 1 hour. Total Ror2 protein levels were analyzed by WB with an anti-Ror2 antibody. (**C**) Total Ror2 protein levels were determined by WB in control or CK1ε CRISPR cells overexpressing Ror2-HA or GST for 48 hours. (**D**) RNA was isolated from control and CK1ε CRISPR cells. Ror2 RNA levels were measured by semi-quantitative RT-PCR. (**E**) Control or CK1ε HEK293T CRISPR were treated with MG132 (MG, 10 μM) or Chloroquine (Chlq, 150 μM) for 6 hours; cells extracts were prepared and Ror2 or Actin were analyzed by WB.

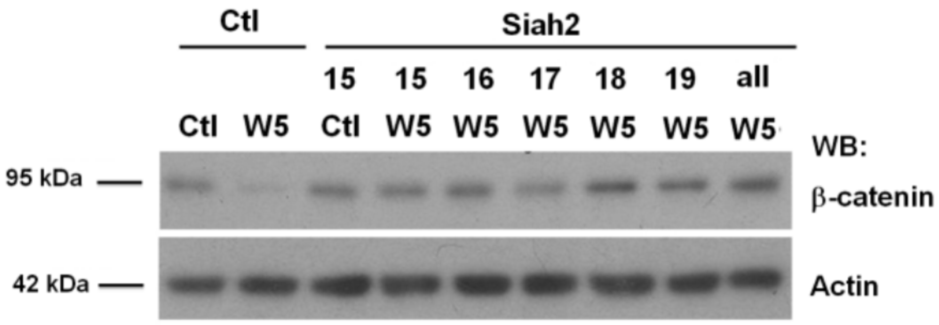

Fig. S12. **Siah2 shRNA prevents β-catenin down-regulation caused by Wnt5a**. HEK293T cells were depleted of Siah2 using a specific shRNA or scrambled. After 48 hours, cells were stimulated with control or Wnt5a-conditioned medium overnight, and β-catenin protein levels were analyzed by WB from total cell extracts.

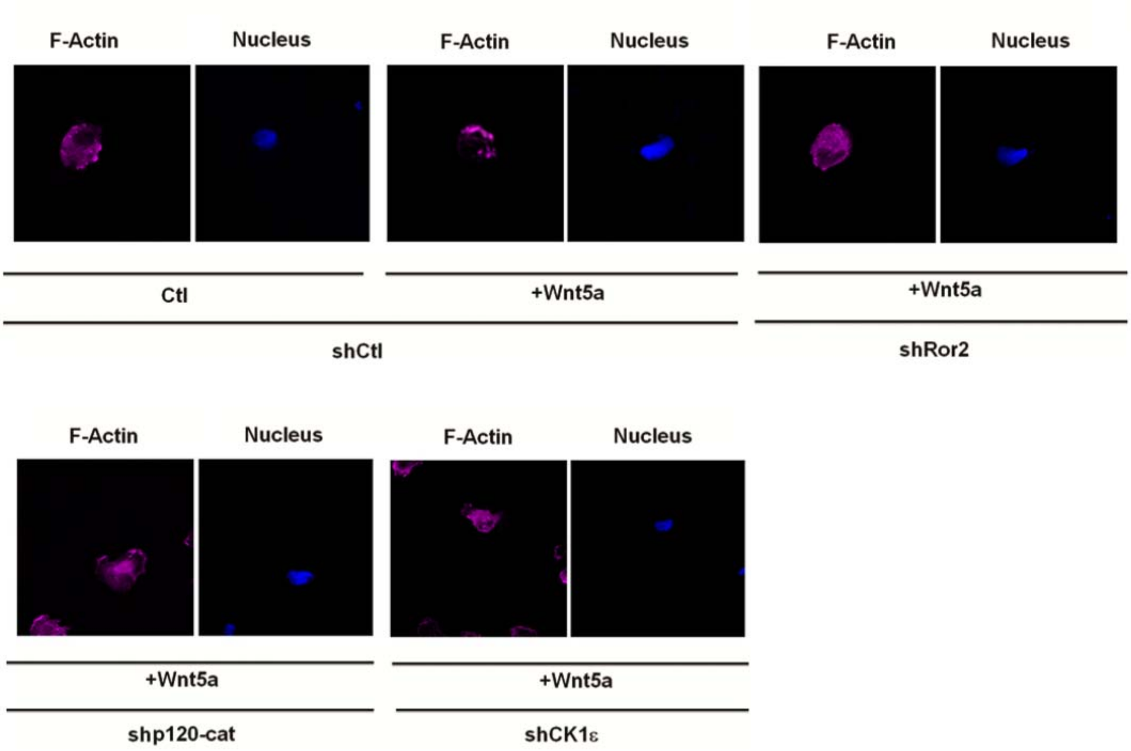

Fig. S13. Ror2, p120-catenin and CK1ε are required for Wnt5a-induced asymmetrical distribution of cortical actin in IEC-18 cells. IEC-18 cells were transfected with the indicated shRNAs and a GFP-expression plasmid; after 24 hours cells were plated on Matrigel for 2h with control or Wnt5a-conditioned medium, fixed and stained for F-Actin and nucleus with Dapi. A GFP-positive cell showing a representative actin staining in the indicated conditions is shown.

Antibody	Supplier	Reference	Assay
Axin	Santa Cruz Biotechnologies	sc-14029	WB
β-Actin	Sigma	A5441	WB
β-catenin	BD Biosciences	610153	WB
CK1γ	Abcam	ab64829	WB/IP
CK1ε	BD Biosciences	610445	WB/IP
CK1δ	Abcam	ab48031	WB
Dvl2	Cell Signaling	3216	WB
EEA1	BD Biosciences	610457	WB
ERK1/2	Cell Signaling	9107	WB
Fz (total)	Santa Cruz Biotechnologies	sc-9169	WB/IP
Fz2	Abcam	ab52565	WB/IP
GST	GE Healthcare	27457701	WB
HA	Roche	11867423001	WB/IP
JNK2	Abcam	ab178953	WB
LRP6	Santa Cruz Biotechnologies	sc-15399	WB
Na^+, K^+ ATPase	Abcam	ab7671	WB
N-cadherin	BD Biosciences	610921	WB/IP
p120-catenin	BD Biosciences	610134	WB/IP
Phospho ERK 1/2 (Thr202/Tyr204)	Cell Signaling	4370	WB
Phospho JNK (Thr183/Tyr185, Thr221/Tyr223)	Millipore	07-175	WB
Phospho p120-catenin (Ser 268)	Santa Cruz Biotechnologies	sc-293000	WB
Phospho LRP5/6 (Thr1490)	Cell Signaling	2568	WB
Phospho Tyrosine	BD Biosciences	610000	WB/IP
PR61ε	Jin et al. 2009		WB
Rac1	BD Biosciences	610650	WB
Ror2	Santa Cruz Biotechnologies	sc-374174	WB/IP
Wnt5a	R&D System	AF645	Wnt5a-specific neutralization

Table S1: List of antibodies used in this work. The table indicates the source, catalogue number and use in Western Blot (WB) or immunoprecipitation (IP) experiments of all the antibodies employed in this article.
