# Supplementary material for: CK1ε and p120‐catenin control Ror2 function in noncanonical Wnt signaling
Source: Mol Oncol. 2018 Mar 14;12(5):611–29. doi: 10.1002/1878-0261.12184 (PMC5928365; doi:10.1002/1878-0261.12184)
Supplement: Supplementary file 16 [file MOL2-12-611-s015.pdf]

Supplementary Information for

CK1ε and p120-catenin control Ror2 function in non-canonical Wnt signaling

Josué Curto, Beatriz Del Valle-Pérez, Aida Villarroel, Guillem Fuertes, Meritxell Vinyoles, Raúl Peña, Antonio García de Herreros and Mireia Duñach

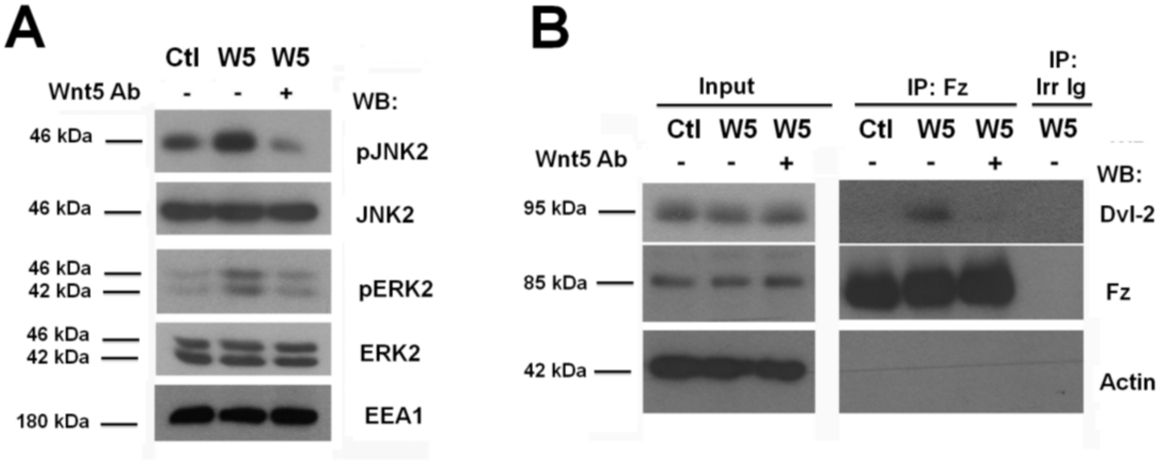

Figure S1

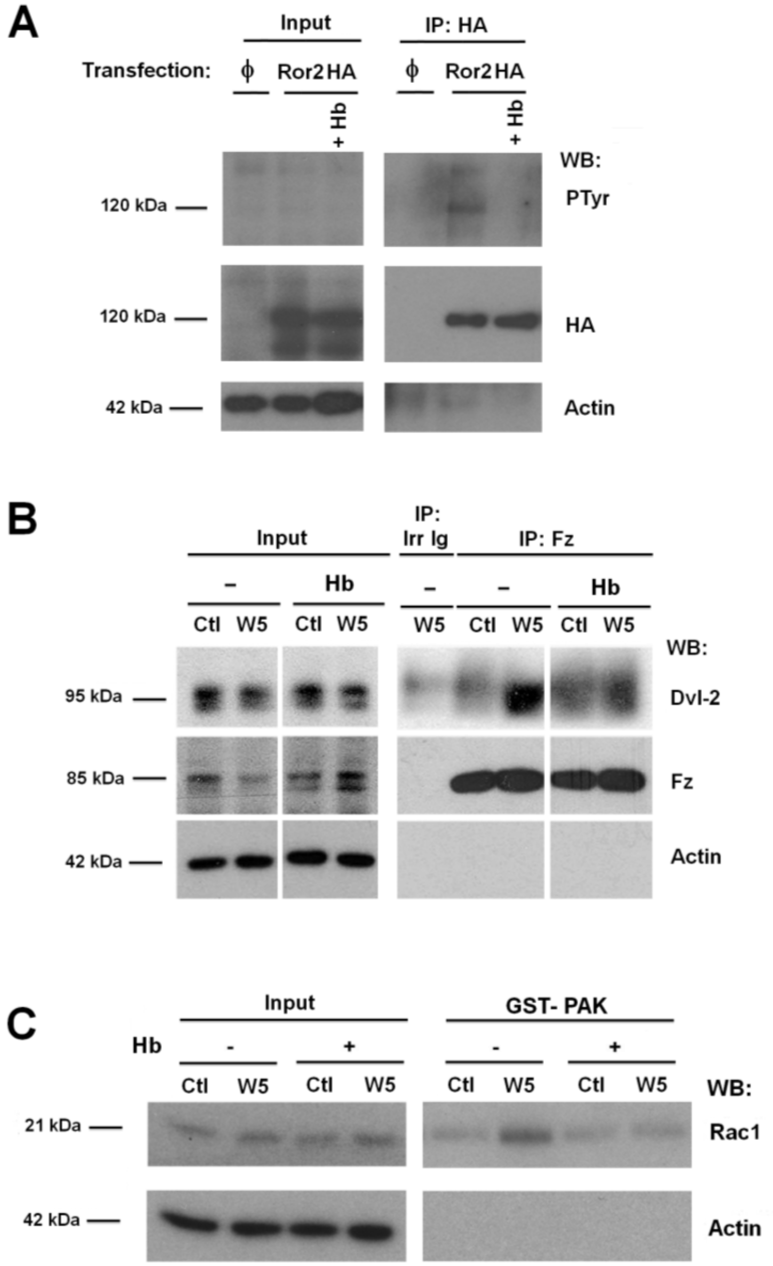
A
Input
IP: HA
Transfection:
ϕ
Ror2 HA
ϕ
Ror2 HA
+ Hb
+ Hb
WB:
120 kDa
PTyr
120 kDa
HA
42 kDa
Actin
B
Input
IP: Irr Ig
IP: Fz
–
Hb
–
–
Hb
Ctl
W5
Ctl
W5
W5
Ctl
W5
Ctl
W5
WB:
95 kDa
Dvl-2
85 kDa
Fz
42 kDa
Actin
C
Input
GST- PAK
Hb
-
+
-
+
Ctl
W5
Ctl
W5
Ctl
W5
Ctl
W5
WB:
21 kDa
Rac1
42 kDa
Actin

Figure S2

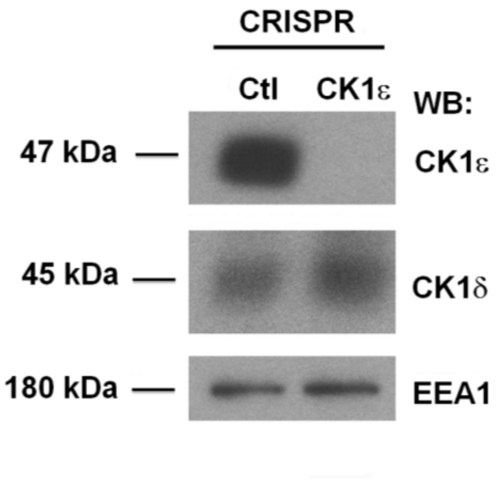

Figure S3

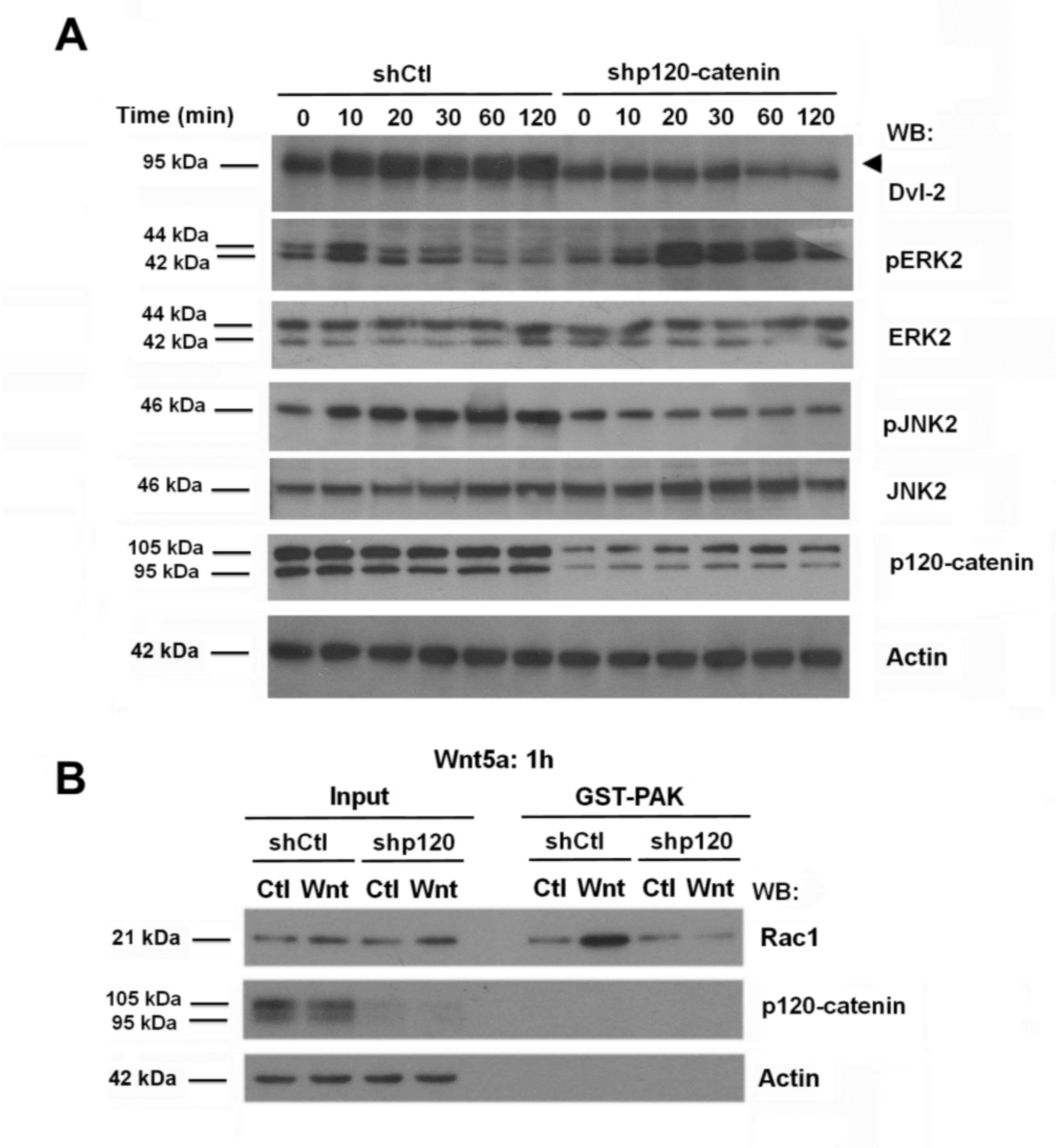
A
shCtl
shp120-catenin
Time (min)
0 10 20 30 60 120 0 10 20 30 60 120
WB:
95 kDa
Dvl-2
44 kDa
42 kDa
pERK2
44 kDa
42 kDa
ERK2
46 kDa
pJNK2
46 kDa
JNK2
105 kDa
95 kDa
p120-catenin
42 kDa
Actin
B
Wnt5a: 1h
Input
GST-PAK
shCtl
shp120
shCtl
shp120
Ctl Wnt Ctl Wnt Ctl Wnt Ctl Wnt
WB:
21 kDa
Rac1
105 kDa
95 kDa
p120-catenin
42 kDa
Actin

Figure S4

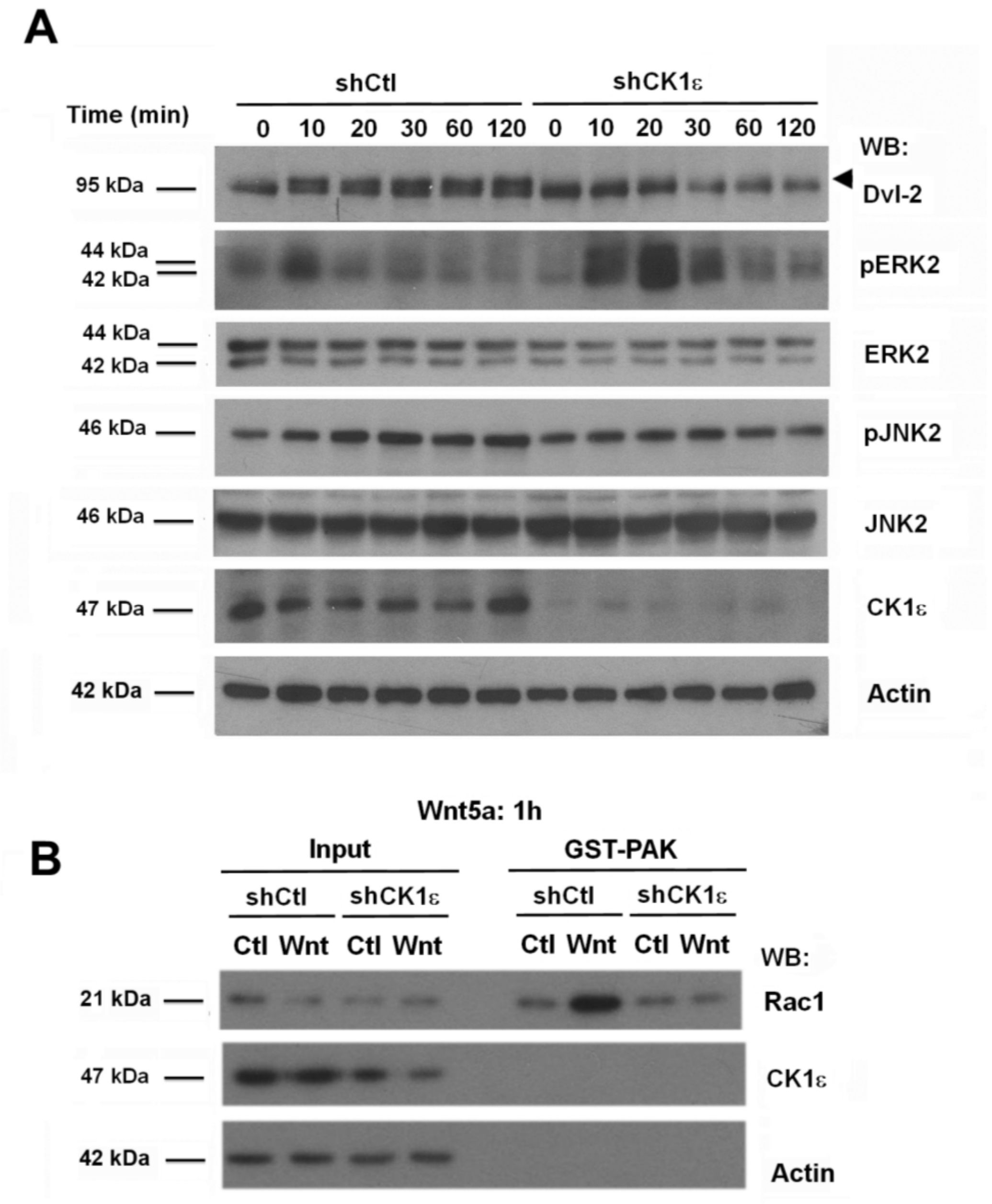
A
shCtl
shCK1ε
Time (min)
0 10 20 30 60 120 0 10 20 30 60 120
WB:
95 kDa
Dvl-2
44 kDa
42 kDa
pERK2
44 kDa
42 kDa
ERK2
46 kDa
pJNK2
46 kDa
JNK2
47 kDa
CK1ε
42 kDa
Actin
Wnt5a: 1h
B
Input
GST-PAK
shCtl
shCK1ε
shCtl
shCK1ε
Ctl Wnt Ctl Wnt Ctl Wnt Ctl Wnt
WB:
21 kDa
Rac1
47 kDa
CK1ε
42 kDa
Actin

Figure S5

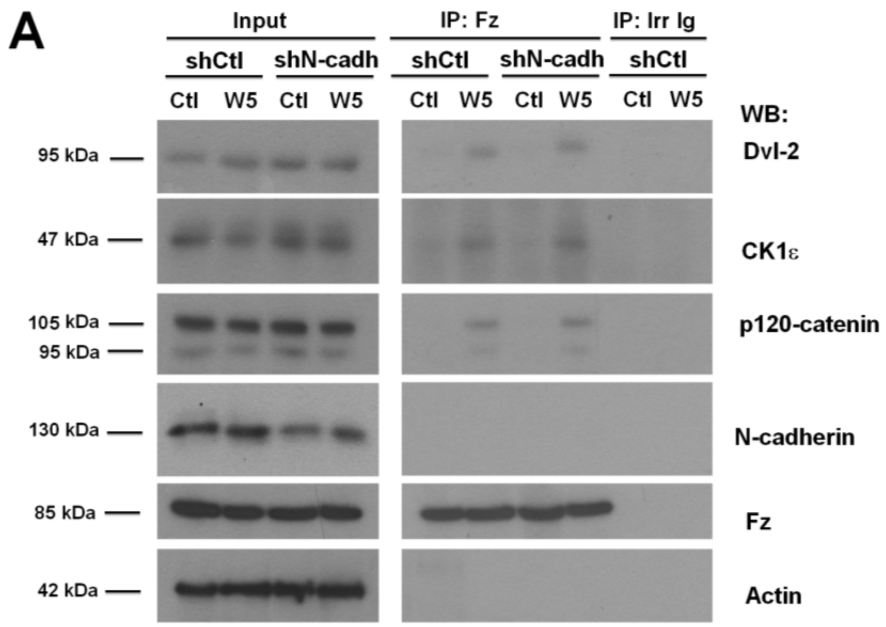

A
Input
IP: Fz
IP: Irr Ig
shCtl
shN-cadh
shCtl
shN-cadh
shCtl
Ctl
W5
Ctl
W5
Ctl
W5
Ctl
W5
Ctl
W5
WB:
95 kDa
Dvl-2
47 kDa
CK1ε
105 kDa
95 kDa
p120-catenin
130 kDa
N-cadherin
85 kDa
Fz
42 kDa
Actin

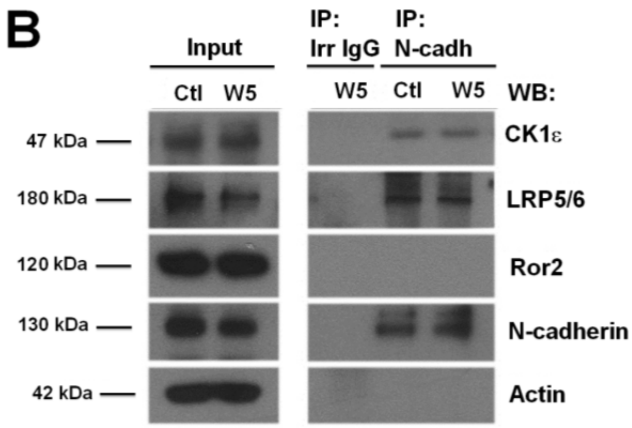

B
Input
IP: Irr IgG
IP: N-cadh
Ctl
W5
W5
Ctl
W5
WB:
47 kDa
CK1ε
180 kDa
LRP5/6
120 kDa
Ror2
130 kDa
N-cadherin
42 kDa
Actin

Figure S6

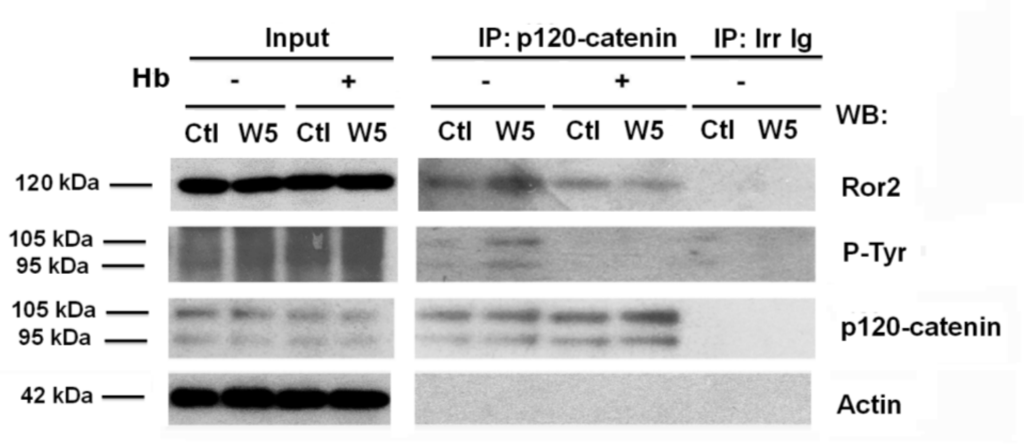
Input
IP: p120-catenin
IP: Irr Ig
Hb
-
+
-
+
-
WB:
Ctl
W5
Ctl
W5
Ctl
W5
Ctl
W5
Ctl
W5
120 kDa
Ror2
105 kDa
95 kDa
P-Tyr
105 kDa
95 kDa
p120-catenin
42 kDa
Actin

Figure S7

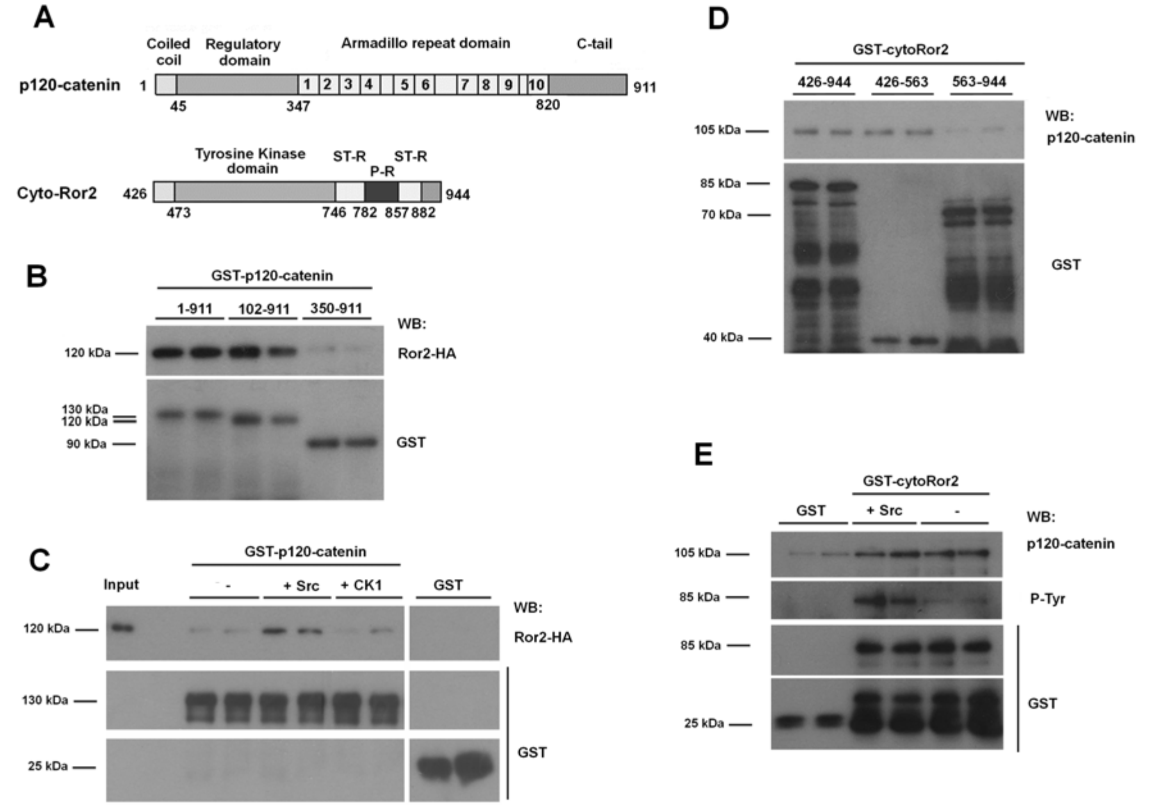
A
Coiled coil
Regulatory domain
Armadillo repeat domain
C-tail
p120-catenin
1
45
347
820
911
Cyto-Ror2
Tyrosine Kinase domain
ST-R
P-R
ST-R
426
473
746
782
857
882
944
B
GST-p120-catenin
1-911
102-911
350-911
WB:
Ror2-HA
GST
120 kDa
130 kDa
120 kDa
90 kDa
C
Input
GST-p120-catenin
-
+ Src
+ CK1
GST
WB:
Ror2-HA
GST
120 kDa
130 kDa
25 kDa
D
GST-cytoRor2
426-944
426-563
563-944
WB:
p120-catenin
GST
105 kDa
85 kDa
70 kDa
40 kDa
E
GST-cytoRor2
GST
+ Src
-
WB:
p120-catenin
P-Tyr
GST
105 kDa
85 kDa
85 kDa
25 kDa

Figure S8

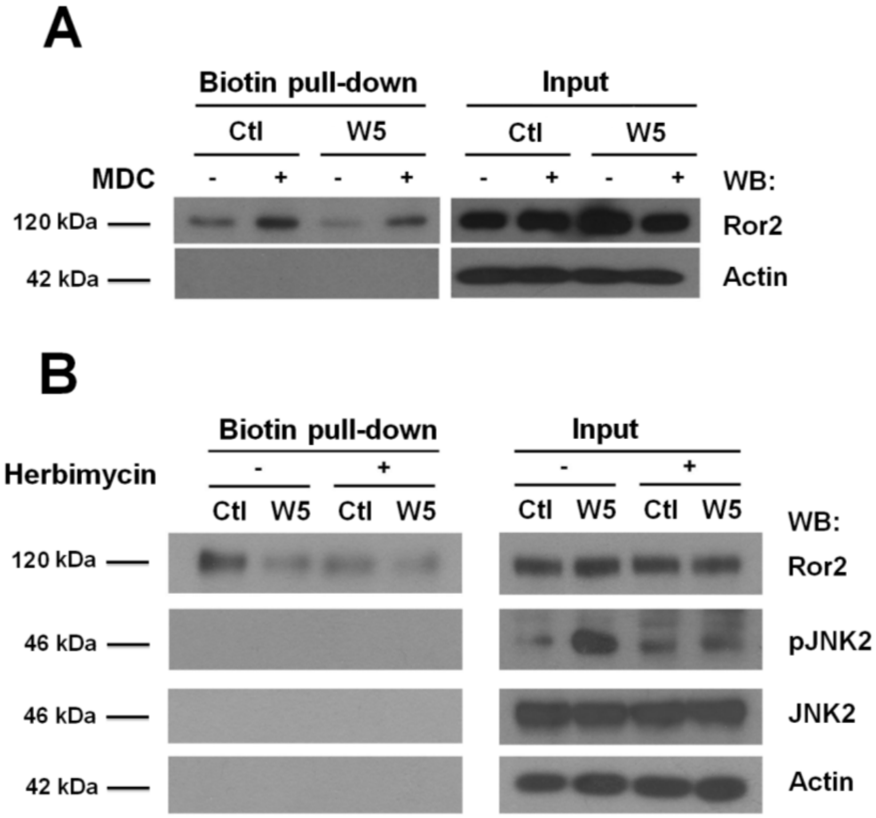

A
Biotin pull-down
Input
Ctl
W5
Ctl
W5
MDC
- + - + - + - +
WB:
120 kDa
Ror2
42 kDa
Actin
B
Biotin pull-down
Input
Herbimycin
- + - +
Ctl W5 Ctl W5
Ctl W5 Ctl W5
WB:
120 kDa
Ror2
46 kDa
pJNK2
46 kDa
JNK2
42 kDa
Actin

Figure S9

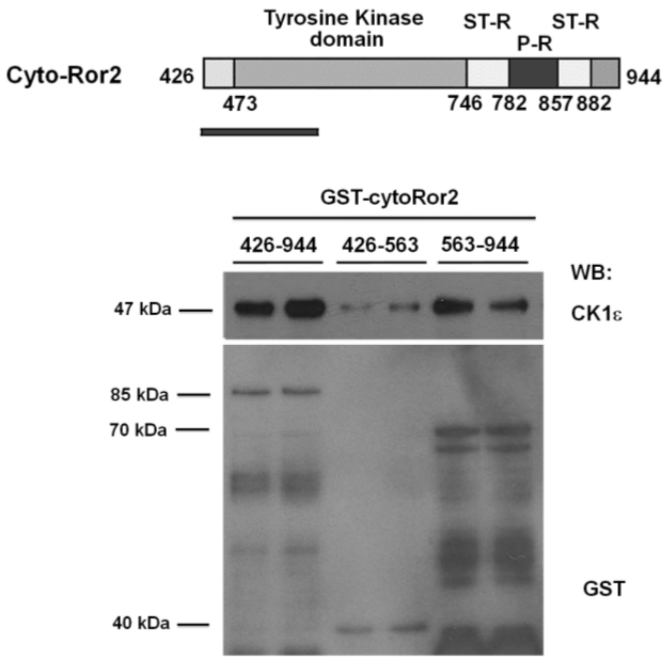
Tyrosine Kinase domain
ST-R
ST-R
P-R
Cyto-Ror2
426
944
473
746
782
857
882
GST-cytoRor2
426-944
426-563
563-944
WB:
47 kDa
CK1ε
85 kDa
70 kDa
GST
40 kDa

Figure S10

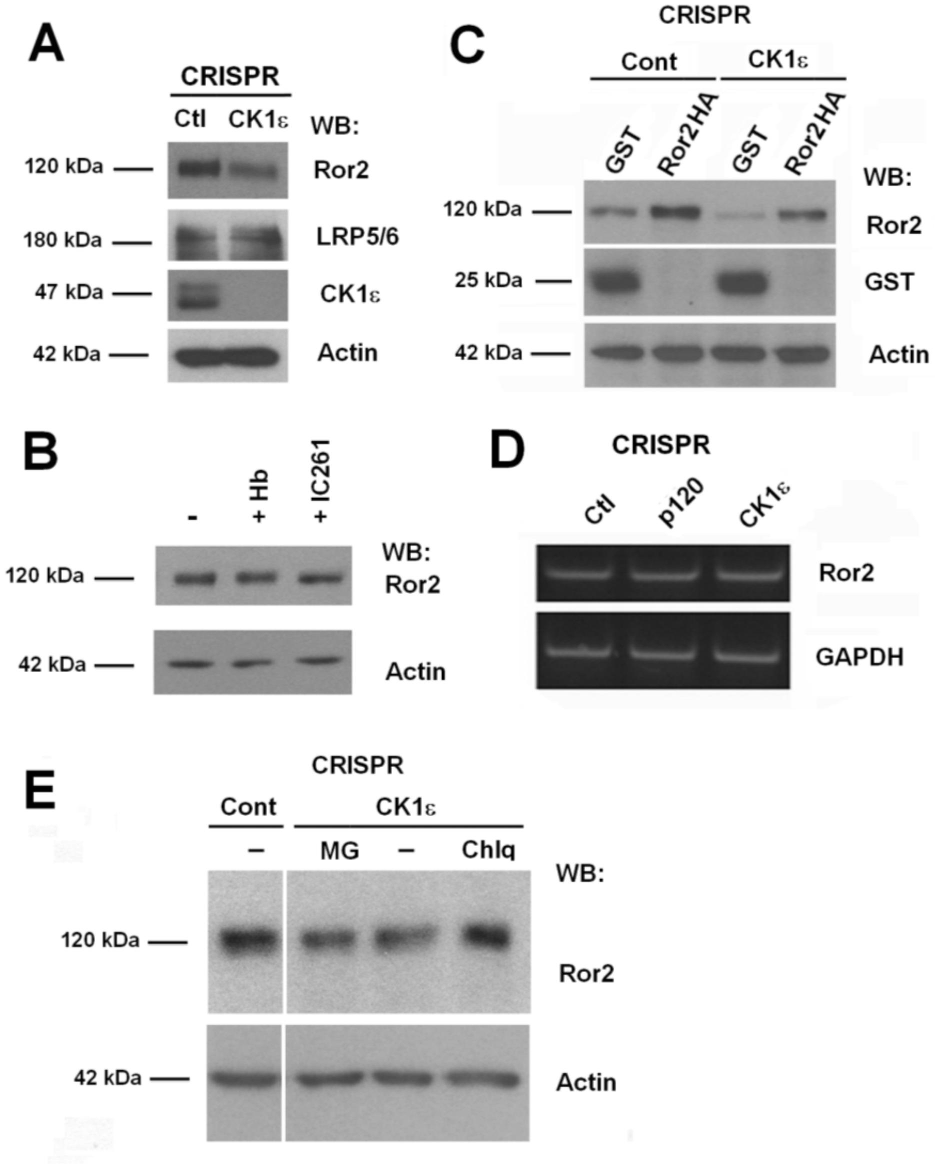
A
CRISPR
Ctl
CK1ε
WB:
120 kDa
Ror2
180 kDa
LRP5/6
47 kDa
CK1ε
42 kDa
Actin
C
CRISPR
Cont
CK1ε
GST
Ror2HA
GST
Ror2HA
WB:
120 kDa
Ror2
25 kDa
GST
42 kDa
Actin
B
-
+ Hb
+ IC261
WB:
120 kDa
Ror2
42 kDa
Actin
D
CRISPR
Ctl
p120
CK1ε
Ror2
GAPDH
E
CRISPR
Cont
CK1ε
–
MG
–
Chlq
WB:
120 kDa
Ror2
42 kDa
Actin

Figure S11

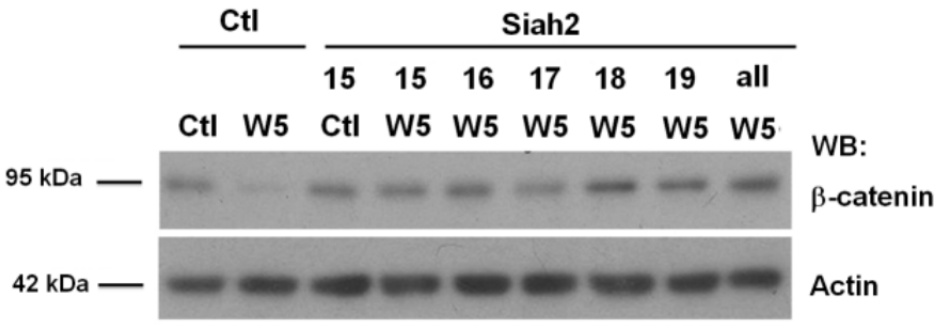
Ctl
Siah2
15
15
16
17
18
19
all
Ctl
W5
Ctl
W5
W5
W5
W5
W5
W5
WB:
95 kDa
β-catenin
42 kDa
Actin

Figure S12

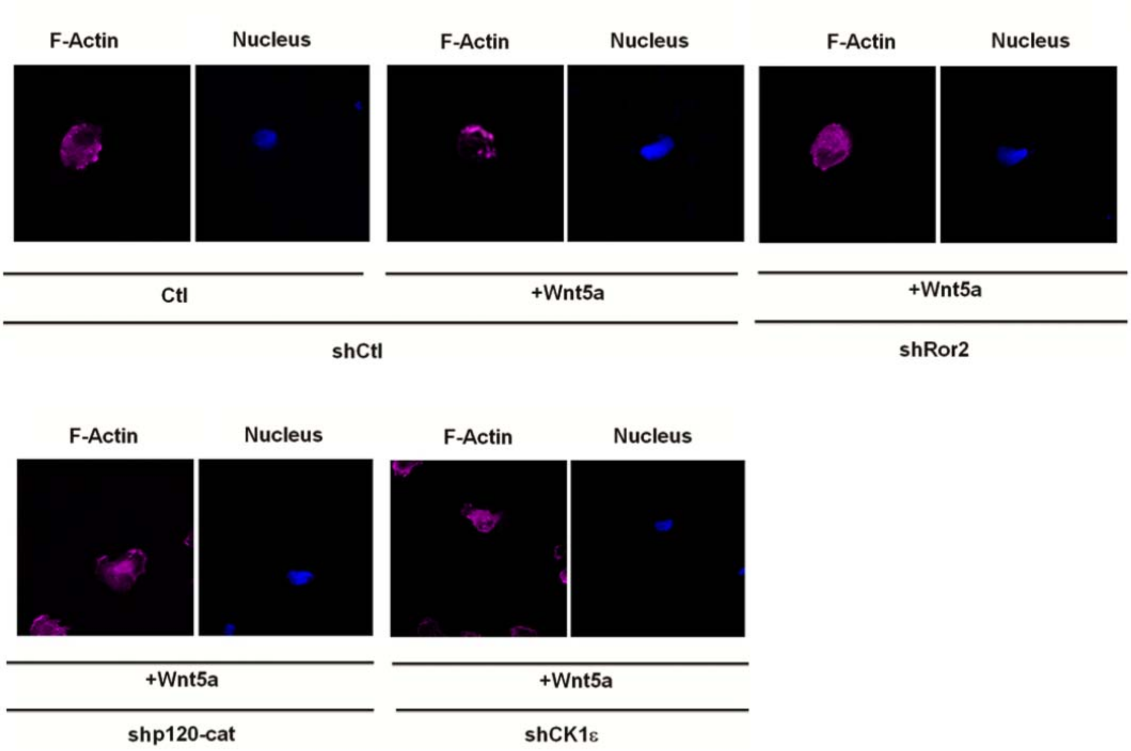
F-Actin
Nucleus
F-Actin
Nucleus
F-Actin
Nucleus
Ctl
+Wnt5a
+Wnt5a
shCtl
shRor2
F-Actin
Nucleus
F-Actin
Nucleus
+Wnt5a
+Wnt5a
shp120-cat
shCK1ε

Figure S13
